# Bi_2_S_3_‐Cu_3_BiS_3_ Mixed Phase Interlayer for High‐Performance Cu_3_BiS_3_‐Photocathode for 2.33% Unassisted Solar Water Splitting Efficiency

**DOI:** 10.1002/advs.202206286

**Published:** 2023-01-16

**Authors:** Subin Moon, Jaemin Park, Hyungsoo Lee, Jin Wook Yang, Juwon Yun, Young Sun Park, Jeongyoub Lee, Hayoung Im, Ho Won Jang, Wooseok Yang, Jooho Moon

**Affiliations:** ^1^ Department of Materials Science and Engineering Yonsei University Seoul 03722 Republic of Korea; ^2^ Department of Materials Science and Engineering Research Institute of Advanced Materials Seoul National University Seoul 08826 Republic of Korea; ^3^ School of Chemical Engineering Sungkyunkwan University Suwon 16419 Republic of Korea; ^4^ SKKU Institute of Energy Science and Technology (SIEST) Sungkyunkwan University Suwon 16419 Republic of Korea

**Keywords:** Cu_3_BiS_3_ photocathode, PEC tandem, solar hydrogen, solar‐to‐hydrogen efficiency, water splitting

## Abstract

To realize practical solar hydrogen production, a low‐cost photocathode with high photocurrent density and onset potential should be developed. Herein, an efficient and stable overall photoelectrochemical tandem cell is developed with a Cu_3_BiS_3_‐based photocathode. By exploiting the crystallographic similarities between Bi_2_S_3_ and Cu_3_BiS_3_, a one‐step solution process with two sulfur sources is used to prepare the Bi_2_S_3_–Cu_3_BiS_3_ blended interlayer. The elongated Bi_2_S_3_‐Cu_3_BiS_3_ mixed‐phase 1D nanorods atop a planar Cu_3_BiS_3_ film enable a high photocurrent density of 7.8 mA cm^−2^ at 0 V versus the reversible hydrogen electrode, with an onset potential of 0.9 V_RHE_. The increased performance over the single‐phase Cu_3_BiS_3_ thin‐film photocathode is attributed to the enhanced light scattering and charge collection through the unique 1D nanostructure, improved electrical conductivity, and better band alignment with the n‐type CdS layer. A solar‐to‐hydrogen efficiency of 2.33% is achieved under unassisted conditions with a state‐of‐the‐art Mo:BiVO_4_ photoanode, with excellent stability exceeding 21 h.

## Graphical abstract

A novel solution‐processed Bi_2_S_3_–Cu_3_BiS_3_ mixed phase is fabricated for photoelectrochemical photocathodes generating high photocurrents and onset potential. By exploiting the unique dual‐sulfur‐source chemistry, unique nanostructure is obtained. The Bi_2_S_3_–Cu_3_BiS_3_ photocathode demonstrates synergetic effects of enhanced light absorption and superior charge transport, enabling 2.33% unassisted solar‐to‐hydrogen conversion efficiency when coupled with Mo:BiVO_4_ photoanode.

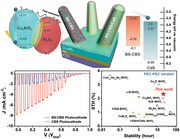

## 1 Introduction

Photoelectrochemical (PEC) water splitting directly converts intermittent solar energy into storable H_2_, which has the highest specific gravimetric energy without generating harmful byproducts.^[^ ^1^, ^2^ ^]^ The potential energy from PEC cells must be greater than the thermodynamically required energy for water splitting (1.23 eV) as well as the overpotential losses occurring at both the anode and cathode to initiate the water splitting reaction.^[^ ^3^ ^]^ In this regard, maximum solar‐to‐hydrogen (STH) conversion efficiency (*η*_STH_) can be achieved with a tandem configuration having stacked dual light absorbers. In this configuration (i.e., the D4‐stacked system), each semiconductor should retain appropriate bandgap (*E*_g_) and band edge positions for the oxygen and hydrogen redox potentials to achieve high *η*_STH_. For example, each semiconductor requires a bottom light absorber (i.e., photocathode) with *E*_g_ of 1.23 eV and top light absorber (i.e., photoanode) with *E*_g_ of 1.84 eV for a theoretical maximum *η*_STH_ of 22.8%. This prediction provides a guideline for selecting absorber materials with desirable optoelectronic properties to maximize the achievable device efficiency.^[^ ^4^ ^]^

Despite the numerous reported studies on improving the *η*_STH_, the limited availability of absorber materials has resulted in low *η*_STH_ for PEC tandem devices with cost‐effective semiconductors. Light absorbers with relatively narrow *E*_g_ (<1.3 eV) values have advantages as the bottom photoelectrodes in tandem cells in that they can harvest a wide range of solar photons; however, the *η*_STH_ based on narrow *E*_g_ semiconductors is limited. For example, only 1.72% *η*_STH_ was reported from PEC tandem cells based on SnS, which is a low‐cost semiconductor with narrow *E*_g_ ≈ 1.23 eV.^[^ ^5^ ^]^ Sb_2_Se_3_, which is another low‐cost light absorber with *E*_g_ ≈ 1.2 eV, exhibited 1.5% *η*_STH_ when coupled with a BiVO_4_ photoanode as the top cell.^[^ ^6^ ^]^ These limited efficiencies were mainly attributed to the low photovoltages from the narrow *E*_g_ bottom photocathodes as the maximum photovoltage is theoretically restricted by the bandgap of the absorber material. The theoretical maximum photovoltage (*V*_ph,max_) is about 0.4 V less than *E*_g_ and is obtained by subtracting the thermodynamic and entropic losses for nonradiative exciton recombination, incomplete light trapping, and re‐radiation, as expressed by the following equation:

(1) $$\begin{array}{c} \begin{array}{c} qV_{\mathrm{oc}}=E_{g}\left(1-T/T_{\mathrm{sun}}\right)-\mathrm{KT}\left[\ln\left(\Omega_{\mathrm{emit}}/\Omega_{\mathrm{sun}}\right)+\ln\left(4n^{2}/I\right)-\;\ln\left(\mathrm{QE}\right)\right] \end{array} \end{array}$$

where *T* and *T*_sun_ are the temperatures of the photovoltaic cell and of the Sun, respectively; *Ω*_sun_ is 6  ×  10^−5^ steradians, *Ω*_emit_ is 4*π*, *n* is the refractive index, *I* is the light concentration factor, and QE is the quantum efficiency factor.^[^ ^7^ ^]^ However, the actual onset potentials of the photocathodes reported so far are significantly lower than *V*_ph,max_. For instance, although the *V*_ph,max_ of the SnS‐based photocathode approaches 0.8 V considering its bandgap, the actual maximum photovoltage is only 0.39 V owing to nonidealities, such as the presence of surface states and defects. Hence, it is essential to mitigate the nonidealities by developing effective techniques, such as surface treatments, interface engineering, and doping.^[^ ^8^, ^9^, ^10^, ^11^ ^]^ Further, higher photovoltages can be attained when large‐*E*_g_ materials are used. However, except for Cu_2_O, there are only a few reported studies on suitable low‐cost and large‐*E*_g_ materials for photocathodes. Thus, it is worth exploring new materials with larger *E*_g_ values to achieve higher photovoltages and eventually high values of *η*_STH_.

Cu_3_BiS_3_ composed of earth‐abundant elements has emerged as an attractive photocathode material exhibiting a high onset potential owing to its large *E*_g_ of 1.86 eV. The theoretical maximum photocurrent density is ≈20 mA cm^−2^ under the assumption of 100% incident photo‐to‐current conversion efficiency (IPCE). Despite the high theoretical photocurrent density, most of the reported Cu_3_BiS_3_ photoelectrodes show poor photocurrent densities. One frequently noted drawback of Cu_3_BiS_3_ is its high conduction band minimum (CBM) edge, resulting in unfavorable band alignment with the common n‐type junction layer of CdS; thus, strategies for lowering the CBM or better counter junction materials should be developed.^[^ ^12^, ^13^ ^]^ Jiang et al. reported lowering the conduction band edge of CdS by doping additional In atoms.^[^ ^14^ ^]^ However, it is believed that an alternative method of reducing band offset without introducing toxic elements is still required. Another possible performance‐limiting factor is low electrical conductivity, which could limit the collection of photogenerated carriers. The formation of appropriate nanostructures based on Cu_3_BiS_3_ can be a feasible solution to overcome such limitations by enhancing the light scattering and decreasing the diffusion length to increase carrier collection.

Herein, we developed a novel synthetic method for in‐situ decoration of Bi_2_S_3_ on Cu_3_BiS_3_ film via one‐step solution processing for successful demonstration of an efficient and stable Cu_3_BiS_3_‐based photocathode for bias‐free solar water splitting. A precursor ink containing a 3:1 stoichiometric amount of Cu:Bi resulted in a single‐phase Cu_3_BiS_3_ film with a dense planar morphology. In contrast, a precursor ink containing excess Bi beyond the stoichiometric amount resulted in simultaneous formation of a Bi_2_S_3_‐Cu_3_BiS_3_ mixed phase with elongated 1D nanorods atop the planar film (e.g., blended Cu_3_BiS_3_). It was shown that the structural similarities between Bi_2_S_3_ and Cu_3_BiS_3_ as well as the unique dual‐sulfur‐sources chemistry enabled the distinctive structure. The 1D nanorods of the mixed phase exhibited enhanced light‐trapping capability, resulting in a decrease in the surface reflectance. Additionally, the Bi_2_S_3_‐Cu_3_BiS_3_ mixed phase allowed better band alignment with the n‐type counterpart by modifying the band structure and enabling facile charge transport via improved electrical conductivity. As a result, the Bi_2_S_3_‐Cu_3_BiS_3_‐based photocathode achieved a high onset potential of 0.9 V_RHE_ with a high photocurrent density of 7.8 mA cm^−2^ at 0 V_RHE_ after additional surface engineering and cocatalyst deposition, which is approximately three times higher than that of the single‐phase Cu_3_BiS_3_ photocathode. Furthermore, an unbiased standalone PEC tandem device was implemented by combining the blended Cu_3_BiS_3_ photocathode with a Mo:BiVO_4_ photoanode. The high photovoltage of the blended Cu_3_BiS_3_ photocathode helped accomplish an overall water splitting reaction with *η*_STH_ of 2.33% under the unassisted condition, maintaining stability for over 20 h.

## 2 Results and Discussion

**Figure 1:**
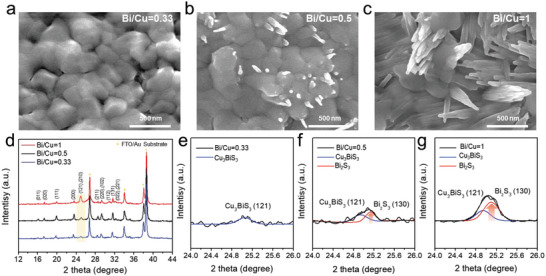
Top‐view scanning electron microscopy (SEM) images of the Cu_3_BiS_3_ films fabricated from different inks with Bi/Cu ratios of a) 0.33, b) 0.5, and c) 1. d) Diffraction patterns of the obtained films. Decoupled diffraction patterns of the peak near 25° for e) Bi/Cu = 0.33, f) Bi/Cu = 0.5, and g) Bi/Cu = 1 films.

The molecular inks for the Cu_3_BiS_3_ films were prepared by dissolving Cu(NO_3_)_2_, Bi(NO_3_)_3_, and thiourea (CH_4_N_2_S, denoted as TU) in 2‐mercaptoethanol (C_2_H_6_OS, denoted as 2MER). The ratios between the Cu and Bi precursors were varied to obtain three different precursor inks with Bi/Cu = 0.33 (stoichiometric ratio), Bi/Cu = 0.5 (moderately excess Bi), and Bi/Cu = 1 (highly excess Bi). Cu_3_BiS_3_ films were then prepared by spin coating each molecular ink, followed by annealing at 300 °C in a N_2_‐filled glovebox. The experimental details are described in the Section 4. Scanning electron microscopy (SEM) was used to characterize the film morphologies based on the initial Bi/Cu ratios in the precursor solutions. Interestingly, each film revealed a distinct microstructure according to its Bi/Cu ratio. The Cu_3_BiS_3_ film fabricated with Bi/Cu = 0.33 ink exhibited a planar morphology (**Figure** **1**a), while the films from the Bi‐excess inks (Bi/Cu = 0.5 and 1) showed elongated nanorods that continuously grew atop the bottom planar film (Figures 1b,c). Longer nanorods were observed when the ink contained more of the Bi precursor (i.e., Bi/Cu = 1).

Regardless of the Bi/Cu ratio, all films exhibited X‐ray diffraction (XRD) peaks of the Cu_3_BiS_3_ orthorhombic phase, with the space group number of 19, and P2_1_2_1_2_1_ without the Cu_x_S impurity phase.^[^ ^15^ ^]^ However, the peak intensity near 25° was enhanced slightly for the increased Bi content (yellow box in Figure 1d). It should be noted that Cu_3_BiS_3_ has distorted tetrahedral BiS_3_ units, with the Bi atoms coordinated to three S and the Bi atom at one vertex of the tetrahedron, resembling the single coordination environment seen in bismuthinite Bi_2_S_3._^[^ ^15^ ^]^ Because of this similarity, it is difficult to distinguish the (130) Bi_2_S_3_ diffraction peak from the (121) Cu_3_BiS_3_ diffraction peak.^[^ ^16^, ^17^ ^]^ The diffraction peaks at 25° for both of the Bi‐excess ink‐driven films were deconvolved into two distinct peaks at 24.9° and 25.13°, which are ascribed to the Cu_3_BiS_3_ and Bi_2_S_3_, respectively, since the Cu_3_BiS_3_ at the lower 2‐theta region has a larger d‐spacing than the Bi_2_S_3_. The intensity of the Bi_2_S_3_ peak increased as more Bi was introduced in the precursor ink. On the other hand, the film obtained with Bi/Cu = 0.33 ink showed only the diffraction peak of the Cu_3_BiS_3_ phase.

**Figure 2:**
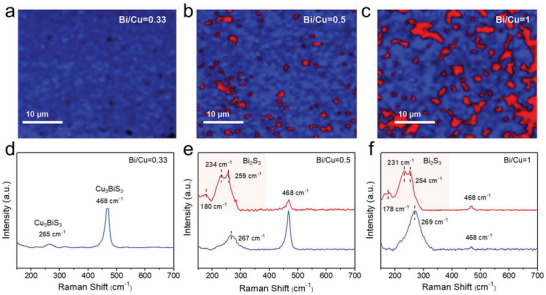
Raman mapping images and spectra of the a,d) Bi/Cu = 0.33, b,e) Bi/Cu = 0.5, and c,f) Bi/Cu = 1 precursor ink‐driven films. The red and blue lines in the Raman spectra represent the red and blue regions in the Raman mapping images, respectively.

Raman mapping images and spectra were obtained to verify the spatial distributions of the Cu_3_BiS_3_ and Bi_2_S_3_ phases (**Figure** **2**a–c). The red and blue lines in the Raman spectra represent the red and blue regions in the Raman mapping images, respectively (Figures 2d–f). It was found that the scanned area of the film fabricated from the Bi/Cu = 0.33 ink was composed of a phase‐pure Cu_3_BiS_3_ (blue), as confirmed by the Raman shift peaks at 265 and 468 cm^−1^ (Figures 2a,d).^[^ ^18^, ^19^, ^20^ ^]^ In contrast, an extra red region was observed along with a blue region in the mapping images of the Bi‐excess ink‐driven films (Figures 2b,c). As shown in Figures 2e,f, the red regions indicate coexistence of the characteristic peaks of the Bi_2_S_3_ (180, 233, and 253 cm^−1^) and Cu_3_BiS_3_ (465 cm^−1^) phases.^[^ ^17^, ^21^ ^]^ The red region extended over a wider range with more Bi in the precursor ink. Therefore, the Raman mapping images demonstrate that the Bi_2_S_3_ phase retains nanorod morphologies and that the formation of the Bi_2_S_3_ nanorods results from the presence of excess Bi. In addition, inductively coupled plasma mass spectrometry (ICP‐MS) measurement was carried out to investigate the final atomic ratio in the films. The Cu_3_BiS_3_ film fabricated with Bi/Cu = 0.33 ink exhibits a ratio of Bi/Cu = 0.29, which is nearly stoichiometric Cu_3_BiS_3_. The samples with Bi/Cu = 1 and 0.5 inks show Bi/Cu = 0.77 and 0.62, respectively, indicating the existence of excess Bi_2_S_3_. The ratio for each element in the final films is listed in Table S1, Supporting Information.

**Figure 3:**
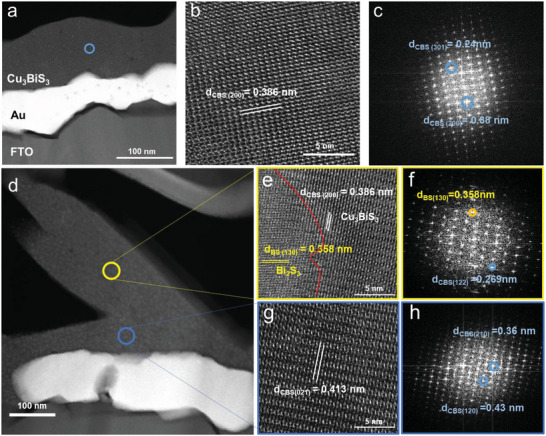
Low‐magnification TEM images of the a) CBS and d) BS‐CBS films. High‐resolution TEM and FFT images of the selected area for b,c) CBS and e–h) BS‐CBS films.

The films from the Bi/Cu = 0.33 and Bi/Cu = 1 inks (denoted hereafter as CBS and BS‐CBS respectively) showing distinct morphologies were investigated by transmission electron microscopy (TEM). **Figure** **3**a,d presents the cross‐sectional low‐magnification TEM images of the films, which are in good agreement with the SEM images (Figure S1, Supporting Information). Cu, Bi, and S atoms were homogeneously distributed in the films, as shown in the EDS elemental images (Figure S2, Supporting Information). An additional high‐resolution TEM (HR‐TEM) was conducted for the deep phase analysis. Figure 3b shows the HR‐TEM image of the CBS film; the lattice spacing of Cu_3_BiS_3_ (d_(200)_ = 0.386 nm) is consistent with that of the orthorhombic Cu_3_BiS_3_. The fast Fourier transform (FFT) image also reveals the single Cu_3_BiS_3_ phase (*d*_(301)_ = 0.24 nm and *d*_(200)_ = 0.38 nm), as shown in Figure 3c. Meanwhile, HR‐TEM was performed on both the nanorods (Figures 3e,f) and bottom planar region (Figures 3g,h) of the BS‐CBS. As expected from the Raman results (Figures 2c,f), two domains are observed in the selected yellow areas of the nanorods, indicating that the two phases of Bi_2_S_3_ (*d*_(130)_ = 0.358 nm) and Cu_3_BiS_3_ (*d*_(200)_ = 0.386 nm) constitute the nanorods. The (130) Bi_2_S_3_ phase can also be recognized by the brightly displayed parts with the Cu_3_BiS_3_ spot in the FFT image (Figure 3f), which is identical to the results of the XRD analysis. Moreover, the boundary of the Bi_2_S_3_ and Cu_3_BiS_3_ phases is not clearly distinguishable, presumably owing to their similar structural properties (Figure 3e). The Bi_2_S_3_ and Cu_3_BiS_3_ are identical orthorhombic structures containing BiS_3_ tetrahedrons that share a similar lattice constant with the Cu_3_BiS_3_ lattice constant (*a* = 7.72 Å), which is twice the c lattice constant for Bi_2_S_3._^[^ ^22^ ^]^ Therefore, the XRD, Raman, and TEM results consistently suggest that the Bi_2_S_3_ crystal was coherently mixed with Cu_3_BiS_3_ owing to their crystallographic similarities, which could minimize the structure‐oriented defects at the interface.

**Figure 4:**
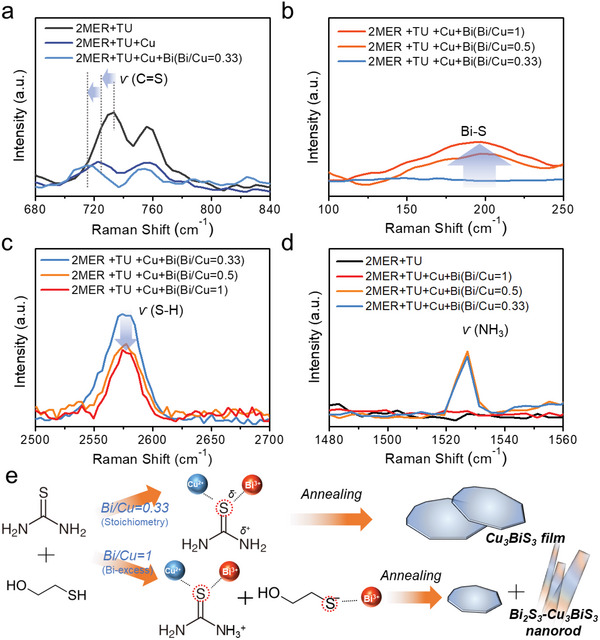
Liquid Raman spectra corresponding to a) S=C stretching for sequential source addition. Liquid Raman spectra for b) Bi–S vibration, c) S–H vibration, and d) NH_3_ stretching mode for the prepared inks. e) Schematic illustration of the film formation mechanism. Addition of a precursor with a stoichiometric ratio (Bi/Cu = 0.33) results in a TU–Cu–Bi molecular complex to produce a Cu_3_BiS_3_ film after annealing. Addition of excess Bi precursor results in Bi_2_S_3_–Cu_3_BiS_3_ mixed‐phase nanorods after annealing through additional complexation with the Bi ions as well as deprotonated thiols (i.e., thiolates) in 2MER.

Understanding the molecular interactions in the precursor inks is essential for understanding the formation mechanism of the unique blended‐phase structure. The preparation of homogeneous solutions containing ternary compound materials at the molecular level is usually challenging owing to the difficulty of dissolving all the precursors in one solvent. Various strategies have thus been reported, including controlling the precursor mixing sequence or adding additional additives and/or stabilizers to dissolve the metal components.^[^ ^23^, ^24^ ^]^ Our precursor ink was simply prepared by sequentially dissolving TU, Cu, and Bi species in 2MER. First, TU was mixed with 2MER, resulting in a colorless clear solution. Upon addition of Cu(NO_3_)_2_ and Bi(NO_3_)_3_ to the solution containing TU, the colorless solution gradually changed to a transparent orange solution (Figure S3a, Supporting Information). However, the addition of Cu(NO_3_)_2_ to the 2MER without TU produced an insoluble white precipitate regardless of the presence of Bi(NO_3_)_3_ (Figure S3b, Supporting Information). This experimental observation implies that the chemical complexation reactions between TU and Cu play a critical role in the preparation of the homogeneous TU‐Cu‐Bi precursor solution without significant segregation. Liquid Raman spectroscopy was performed for the 2MER, 2MER+Cu(NO_3_)_2_, and 2MER+TU+Cu(NO_3_)_2_ containing solutions. A strong peak was observed at 475 cm^−1^ in the case of the solution containing a Cu precursor without TU, which is attributed to the polymerization of the 2MER‐Cu complex (Figure S4, Supporting Information). In contrast, the corresponding peak was barely observable in the TU‐containing solution, while the symmetric stretching vibration of C=S in the TU was slightly red‐shifted from 733 to 722 cm^−1^ (**Figure** **4**a). It should be noted that TU has been used as both a sulfur source and metal‐ion complexing stabilizer in this work to ensure dissolution of the metal components.^[^ ^23^, ^24^, ^25^ ^]^ For TU‐metal complexation, two possible coordination mechanisms have been reported through bonding to either nitrogen or sulfur of the TU. Since the NH_2_ bending modes (at 3200 and 3334 cm^−1^) exhibit negligible changes upon addition of the Cu precursors (Figure S5, Supporting Information), the interactions between the metal ions and sulfur in TU is expected, resulting in a higher electron density developed for the sulfur atom.^[^ ^26^, ^27^ ^]^ The enhanced delocalization of the lone‐pair electrons leads to reduced C=S bond strength and peak shifting toward lower wavelengths.^[^ ^5^, ^24^ ^]^ The addition of the stoichiometric amount of Bi precursor to the TU‐Cu solution additionally shifts the C=S vibration to a lower wavenumber (at 715 cm^−1^, Figure 4a), suggesting that TU acts as a stabilizer to prevent 2MER‐Cu precipitation by the formation of the TU–Cu–Bi complex.

Liquid Raman spectra of the final three precursor inks prepared with the same mixing sequence described above are compared. A broad peak at 200 cm^−1^ is observed, which increases as more Bi is included in the precursor ink, while no peak is seen for the Bi/Cu = 0.33 ink (Figure 4b). This new peak is assigned to the Bi‐S bonding by considering the similarly positioned Raman peak of the A_1g_ mode for the Bi_2_S_3_ film (Figure S6, Supporting Information).^[^ ^17^ ^]^ The Bi_2_S_3_ film was fabricated using inks composed of Bi and TU without Cu by an identical preparation procedure. The corresponding XRD and SEM information are shown in Figure S7, Supporting Information, and the nanorod morphology of the crystalline Bi_2_S_3_ is observed. It should be noted that the S atom of the Bi—S bond originates from the 2MER and not the TU, as the C=S vibration in TU shows negligible differences between the three final solutions upon addition of the excess Bi atoms (Figure S8, Supporting Information). Meanwhile, when excess Bi is contained in the precursor ink, the intensity of the S—H stretching band at 2576 cm^−1^ diminishes slightly (Figure 4c), whereas that of the NH_3_ stretching mode (≈1530 cm^−1^) manifests (Figure 4d).^[^ ^28^ ^]^ These liquid Raman analyses clearly show that the excess Bi deprotonates the thiol (S—H) in 2MER by the formation of NH_3_^+^ (as a result of the protonation of NH_2_ in TU) and reacts with the thiolate (C—S^−^). Interestingly, our 2MER‐TU solvent system resembles other thiol‐amine solvent systems where the amine deprotonates thiol to release thiolate anions.^[^ ^29^ ^]^ However, it is clearly different from the previously reported thiolate‐amine‐mediated dissolution of the metal species because the TU can also be a sulfur source. For the first time, we elucidate the role of TU with 2MER in dissolving a metal species, whereas studies on the previously developed alkahest solution focused on the role of the thiolate nucleophile.^[^ ^29^ ^]^ This observation clearly suggests that two different sulfur sources in the precursor inks induce the unique morphology with the mixed phases of the Bi_2_S_3_ and Cu_3_BiS_3_. The formation of the TU‐Cu‐Bi complex in the Bi/Cu = 0.33 ink eventually becomes a planar Cu_3_BiS_3_ film after spin coating and annealing; the S in TU is the sulfur source for the Cu_3_BiS_3_. On the other hand, it is assumed that the excess Bi cations react to the thiolate in 2MER, to form the Bi_2_S_3_‐related nanorods (Figure 4e). Thus, it is shown that the formation of the Bi‐thiolate complex in conjunction with the coexisting TU–Cu–Bi complex is the origin of the unique Bi_2_S_3_–Cu_3_BiS_3_ mixed‐phase 1D nanorods. In addition, Cu_3_BiS_3_ has a lower Gibbs formation energy (−238.05 kJ mol^−1^) than Bi_2_S_3_ (−136.8 kJ mol^−1^) under the standard states, which allows Cu_3_BiS_3_ to be formed first and followed by the nanorods, allowing the unique morphology of the nanorods grown on the planar films.^[^ ^30^ ^]^

**Figure 5:**
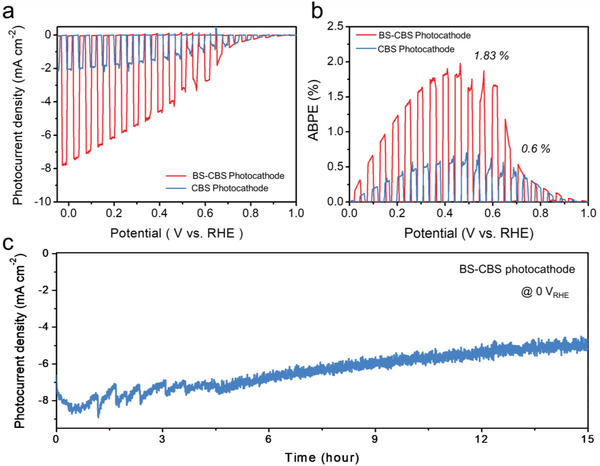
a) *J–V* curves and b) ABPE of both the CBS and BS‐CBS photocathodes in a configuration of pt‐TiO_2_/CdS/Cu_3_BiS_3_‐based film/Au/FTO with the size of 0.15cm^2^ under simulated solar AM 1.5G irradiation in 0.5 m K‐Pi (pH 7). c) Photocurrent density versus time curve for the BS‐CBS photocathode under 0 V_RHE_.

Both BS‐CBS‐ and CBS‐based photocathodes were fabricated in the configuration of FTO/Au/Cu_3_BiS_3_/CdS/TiO_2_/Pt. The CdS, TiO_2_, and Pt were consequently deposited on the absorbing layer via chemical bath deposition, atomic layer deposition, and sputtering methods, respectively (Figure S9, Supporting Information). A linear sweep voltammogram (LSV) was performed to examine the PEC performances of the Cu_3_BiS_3_‐based photocathodes using a three‐electrode configuration under 1 sun illumination (AM 1.5 G). Incomplete passivation can lead to a dark current during PEC performance test under the dark condition due to a direct contact between the Pt co‐catalyst and the conductive substrate. However, our *J–V* curves show no dark current, thus it can be considered that our nanostructures are well‐passivated. The BS‐CBS photocathode generates a higher photocurrent density of nearly 7.8 mA cm^−2^ at 0 V_RHE_ than the CBS photocathode, which provides only 2 mA cm^−2^ (**Figure** **5**a). Figure S10, Supporting Information shows the IPCE analysis conducted on the photocathodes at 0 V_RHE_. The integrated photocurrent densities for the CBS and BS‐CBS photocathodes obtained from the IPCE values are well matched with the LSV measurements. Photoluminescence (PL) spectroscopy was conducted to clarify the bandgap of Cu_3_BiS_3_. The PL signal can be analyzed to infer the bandgap through a phenomenon in which excited carriers fall from the excited state to the ground state. The PL spectrum shows a peak around 655 nm, suggesting that it has a band gap of about 1.89 eV (Figure S11, Supporting Information). The dominant wavelength region for converting light into current on both photocathodes is 300–600 nm, showing that the BS‐CBS film exhibits a much higher IPCE than the CBS photocathode. However, this value indicates that there is still room for improvement of the photocurrent, especially in the 500–600 nm wavelength region. Although the CBS thin film can absorb photons having wavelength up to 655 nm, the IPCE values near the absorption edge are generally low due to the following reasons: 1) the light absorption coefficient near the absorption edge is relatively low and 2) the low‐energy photons can penetrate deeper in the light absorber so that the generated minority carriers have to travel a longer distance. For these reasons, several strategies have been developed to enhance the IPCEs near the absorption edge, such as heteroatom doping or inserting dipole layer, etc.^[^ ^30^, ^31^ ^]^ Both photocathodes have high onset potentials of 0.9 V_RHE_ originating from their wide *E*_g_, which clearly demonstrates the possibility and feasibility of the efficient tandem device comprising a Cu_3_BiS_3_‐based photocathode. The applied bias photon‐to‐current efficiency (ABPE) was calculated from the obtained *J*–*V* curves. The BS‐CBS photocathode exhibits 1.83% at 0.45 V_RHE_, which is three times that of the CBS photocathode (0.6% of the ABPE, Figure 5b). The highest ABPE is observed in such a high potential region (0.45 V_RHE_), which is usually the region in which the PEC tandem device is driven. The BS‐CBS photocathode was found to have an appreciable stability of 15 h at 0 V_RHE_, with nearly 70% retention of its initial current (Figure 5c). After the stability test, its surface structure became coarse and porous (Figure S12, Supporting Information). To provide more evidence regarding the degradation mechanism, stability test with extended period (≈20 h) has been further performed. Figure S13, Supporting Information is the *J–V* curve for the BS‐CBS photocathode after 20 h stability test at 0 V_RHE_ under light, showing much degraded performance compared to the initial value. Despite such degradation of the performance, XRD data in Figure S14, Supporting Information show almost unchanged BS‐CBS peaks, indicating negligible decomposition of BS‐CBS. However, the X‐ray photoelectron spectroscopy (XPS) results before/after the stability test clearly show the dissolution of TiO_2_ layer (Figure S15a, Supporting Information). Before the stability test of the BS‐CBS photocathode, the Ti 2P_3/2_ peak (at 459.2 eV) and the 2P_1/2_ peak (at 464.9 eV) with spin–orbital‐coupling‐induced peak separation of 5.7 eV were attributed to the distinct Ti^4+^ chemical state in TiO_2_. However, after the stability test, the chemical states of Ti were rarely observed. Furthermore, the chemical states of Cu before the stability test were hardly observed due to the TiO_2_ layer with a thickness of ≈35 nm (Figure S15b, Supporting Information). After the stability test, the spectra of the Cu 2p_3/2_ (at 932.20 eV) peak and the 2p_1/2_ peak (at 951.96 eV) can be observed. The doublet separation is 19.8 eV which is assigned to the Cu^+^ in Cu_3_BiS_3_, indicative of dissolved TiO_2_. It is well known that the TiO_2_ overlayer enhances the charge separation as a driving force induced by additional band bending (Figure S16, Supporting Information). Therefore, it seems plausible that the dissolution of TiO_2_ resulted in a rough and porous surface structure and degradation in the BS‐CBS photocathode (Figure S12, Supporting Information). The performance of the BS‐CBS photocathode was systematically compared with that of the Cu‐containing wide‐*E*_g_‐material‐based photocathodes (**Table** **1**). Except for Cu_2_O, which has a long history of development, our BS‐CBS photocathode shows the highest performance in all respects.

**Table 1:** Performance comparison of wide‐*E*_g_‐material‐based photocathode containing Cu‐based low‐cost materials

Photocathode configuration	*J*_SC_ [mA cm^−2^]	Onset potential [V_RHE_]	ABPE [%]	Stability [retention%]	Ref.
BS‐CBS/CdS/TiO_2_/Pt	−7.8 @ 0 V_RHE_	0.9	1.83	15 h (70%)	This work
Cu_3_BiS_3_/In_2_S_3_/Pt	−0.11 @ 0 V_RHE_	0.6	N/A	1 h (90%)	[19]
Cu_3_BiS_3_/CdS/TiO_2_/Pt	−7 @ 0 V_RHE_	0.9	1.7	10 h	[18]
Cu_2_S/CdS/TiO_2_/RuO_x_	−7 @ −0.3 V_RHE_	0.48	N/A	3 h 20 min (90%)	[44]
Cu_2_O/Ga_2_O_3_/TiO_2_/RuO_x_	−8.6 @ 0 V_RHE_	0.92	3.6	1 h (97%)	[45]
CuFe_2_O_4_	−1.82 @ 0.4 V_RHE_	1.05	N/A	300 s	[46]
Cu_2_O/Ga_2_O_3_/TiO_2_/RuO_x_	−10 @ 0 V_RHE_	1	NA	120 h (80%)	[10]
Co‐CuBi_2_O_4_/TiO_2_	−0.3 @ 0.5 V_RHE_	0.9	0.075	N/A	[47]

To elucidate the origin of the superior photocurrent density of the BS‐CBS, we first performed ultraviolet/visible light (UV–vis) spectroscopy to investigate the optical properties of both films. A lower surface reflectance of the BS‐CBS film was observed compared to the CBS film, which agrees with the photographs of the film surfaces, showing shinier (more reflective) surface of the CBS film (Figure S17, Supporting Information). The total reflectance was obtained as the sum of the specular and diffuse reflectances. The decrement of the reflection presumably originated from the nanorod morphology, which led to internal light scattering. With the assumption that the reflected light is absorbed with 100% absorber photon‐fito‐current conversion efficiency, the photocurrent density losses from the reflectances on both films under wavelengths in the 300–800 nm range reach 5.6 mA cm^−2^ for the CBS film and 4.2 mA cm^−2^ for the BS‐CBS film (Figure S18, Supporting Information). The low photocurrent density loss with less reflectance indicates that the BS‐CBS structure exhibits an optical enhancement factor of 1.33 (i.e., proportion of absorbed light amount of the BS‐CBS to CBS).

**Figure 6:**
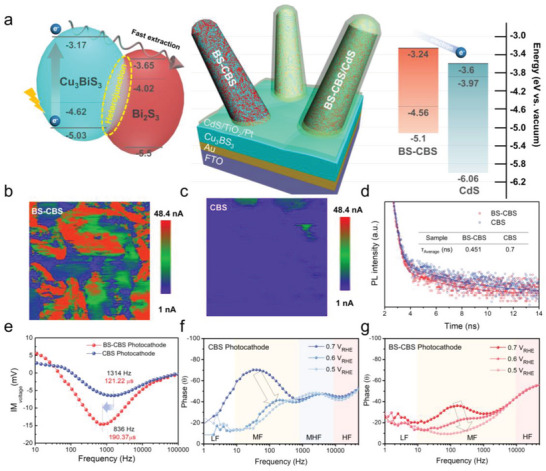
a) Schematic of the BS‐CBS photocathode with band diagram showing the relative energy positions of each of the semiconductors calculated from UPS. Current mappings for the b) BS‐CBS and c) CBS films by c‐AFM measurements. d) Time‐resolved photoluminescence emission decay spectra of the CBS and BS‐CBS films on the Au quenching layer. e) Bode plot of the IMVS for BS‐CBS‐ and CBS‐based photocathodes. Bode plots obtained from EIS measurements of the f) CBS and g) BS‐CBS photocathodes.

However, photocurrent improvements cannot be entirely explained by the better light properties alone considering that the enhancement factor in the photocurrent density for the BS‐CBS photocathode is 3. Another possible explanation for the enhanced photocurrent is the improved electrical properties. Various techniques, such as heteroatom doping, adjusting the crystallographic orientations, and implementation of a surface conduction layer, have been reported to enhance the electrical properties to achieve higher photocurrents.^[^ ^3^, ^32^ ^]^ It should be noted that Bi_2_S_3_ has a higher electrical conductivity (1.087 Ω^−1^ cm^−1^)^[^ ^33^ ^]^ than Cu_3_BiS_3_ (0.03 Ω^−1^ cm^−1^)^[^ ^12^ ^]^ at room temperature, resulting in better carrier mobility. Therefore, Cu_3_BiS_3_ embedded with Bi_2_S_3_ is expected to provide effective charge transport through the nanorods (**Figure** **6**a). Conductive atomic force microscopy (c‐AFM) was conducted to investigate the charge carrier transport in the nanostructured photoelectrodes. Figures 6b,c show the surface current mappings of the CBS and BS‐CBS films, respectively. The BS‐CBS film displays a higher level of current than the CBS film, as represented by the bright red regions along the nanorods. This observation supports the fact that carriers are readily collected through the Bi_2_S_3_‐containing nanorods owing to the improved electrical conductivity. The normalized IPCE spectra (Figure S19, Supporting Information) show the higher efficiency of the low‐energy (long wavelength > 500 nm) photons in the BS‐CBS photocathode than the CBS photocathode, indicating the enhanced carrier collection due to the improved electrical properties and/or nanostructure effects.

Time‐resolved photoluminescence (TRPL) measurements were further investigated to evaluate the photoexcited charge carrier extraction of both films with the configuration of FTO/Au/CBS or BS‐CBS. The TRPL in which all spectra were fitted with triexponential functions is given by Equation (2), with the lifetime being defined by Equation (3)

(2) $$f\left(t\right)=A_{1}\exp(-t/\tau_{1})+A_{2}\exp(-t/\tau_{2})+A_{3}\exp(-t/\tau_{3})$$

(3) $$\tau =\sum A_{i}\tau_{i}^{2}/\sum A_{i}\tau_{i}$$

where *τ*_1_, *τ*_2_ are the fast decay lifetimes and *τ*_3_ is the slow decay lifetime, with weight ratios of *A*_1_, *A*_2_, and *A*_3_, respectively. The fitted results are shown in Figure 6d, and Table S2, Supporting Information summarizes the fitting parameters. The fast decay component is correlated with the carrier decay time influenced by the defects (e.g., such as S vacancies or anti‐sites), while the slow decay component is correlated with the radiative interband recombination that decreases when the photogenerated charges are extracted toward the Au layer.^[^ ^34^, ^35^, ^36^ ^]^ The BS‐CBS film revealed a much shorter average lifetime (*τ*_ave_ = 0.451 µs) than the CBS film (*τ*_ave_ = 0.7 µs). This reduced average lifetime of the BS‐CBS film is mainly attributed to the low *τ*_3_ (Table S2, Supporting Information), indicating fast extraction of the photogenerated charge carriers through the Au quenching layer. Therefore, in conjunction with the c‐AFM results, we can assume that the mixed phase of the BS‐CBS enables the superior charge transport behavior, which in turn affects the photocurrent of the photocathode when utilized as an absorber layer.

A CdS layer is a common n‐type counterpart in many chalcogenide photocathode materials.^[^ ^18^, ^37^, ^38^ ^]^ However, the cliff‐off mismatch in band alignment between the Cu_3_BiS_3_ and CdS has been noted as a performance‐limiting factor.^[^ ^13^, ^18^ ^]^ The band positions of the CdS, CBS, BS‐CBS, and Bi_2_S_3_ were investigated by ultraviolet photoelectron spectroscopy (UPS, Figure S20, Supporting Information). As shown in Figure 6a, Cu_3_BiS_3_ with Bi_2_S_3_ forms an appropriate interfacial band alignment, such as a type‐II heterojunction, which causes a built‐in electric field at the interface.^[^ ^39^, ^40^, ^41^ ^]^ The built‐in electric field enhances charge separation and suppresses recombination. Particularly, this type of junction is formed in the nanorods of the BS‐CBS film located atop the film; thus, additional charge transport facility is expected at the surface of the film. Furthermore, the BS‐CBS/CdS junction has better alignment than the CBS/CdS junction owing to the slightly lowered CBM level, which enhances the charge separation.^[^ ^42^ ^]^ It should be noted that BS‐CBS‐based photocathode was fabricated by sequentially depositing CdS, TiO_2_ overlayers, and Pt co‐catalyst. The CdS layer was formed by the CBD and the TiO_2_ layer and Pt was deposited by the ALD and sputtering, respectively. It is well known that those methods enable the conformal deposition of interlayers on complex structures. Hence, it is expected that interfacial recombination can be minimized by using adequate deposition techniques for appropriate materials to establish proper band alignment and reduce the overpotential. Intensity‐modulated photovoltage spectroscopy (IMVS) analysis is a useful technique for investigating the photogenerated carrier dynamics in relation to interfacial recombination in fully operating PEC devices. IMVS analysis determines the modulation of the photovoltage that is measured simultaneously with a small sinusoidal modulation of the light intensity under open‐circuit conditions (Figure 6e). The peak at high frequency (≈ 1 kHz region) in the Bode plot is the minimum value of the imaginary photovoltage that provides the characteristic rate constant for surface charge recombination (*k*_rec_) proportional to the characteristic frequency(*f*_rec_), whereas the characteristic time constant (*τ*_rec_) can be estimated by Equation (4) below

(4) $$\tau_{\mathrm{rec}}=\left(1/k_{\mathrm{rec}}\right)=\left(1/2\pi f_{\mathrm{rec}}\right)$$

In comparison to the CBS photocathode, the peak of the BS‐CBS photocathode shifts toward the low‐frequency region, indicating an increase in *τ*_rec_ from 121.22 to 190.3 µs. This increased *τ*_rec_ suggests that the surface charge recombination is remarkably suppressed at the surface of the BS‐CBS photocathode under working conditions. Thus, it can be speculated that the enlarged surface area associated with the nanorod structure enhances the charge transport, instead of increasing interface recombination, because of the well‐matched interface between the p‐type CBS and n‐type CdS and TiO_2_. To further identify the carrier dynamics in both the CBS and BS‐CBS photocathodes, electrochemical impedance spectroscopy (EIS) was performed in the range of 50 kHz to 0.5 Hz under 1 sun irradiation. Figure S21, Supporting Information shows the Nyquist plots of the CBS and BS‐CBS photocathodes. A Voight‐type equivalent circuit model was used to fit the curves, which is the serial association of a series of RC parallel elements and provides an accurate description of the electrochemical reactions that fit the obtained EIS data. The curves were fitted with a series resistance (*R*_s_), three serial pairs of parallel combinations of resistance and capacitance for high (*R*_HF_, CPE_HF_), middle (*R*_MF_, CPE_MF_), and low (*R*_LF_, CPE_LF_) frequencies. For convenience, the three deconvolved semicircles (*R*_HF_ with CPE_HF_, *R*_MF_ with CPE_MF_, and *R*_LF_ with CPE_LF_) are distinctly displayed as red, yellow, and white regions, respectively (Figure 6g and Figure S21b, Supporting Information). Interestingly, as shown in Figure 6f and Figure S21a, Supporting Information, the CBS photocathode was fitted with additional components in the middle‐to‐high frequencies (MHFs, @2–3 kHz, *R*_MHF_, CPE_MHF_, blue region). It has been suggested that the RC element at low frequency (LF, 1–10 Hz) is influenced by the charge transfer reactions (i.e., HER), along with the capacitance of the electrode/electrolyte interface. It is also speculated that the RC components at the highest frequency depend on artifacts (e.g., interference by the reference electrode or ohmic resistance from electrolytes, wires, contacts, etc.) regardless of the semiconductor characteristics.^[^ ^43^ ^]^ The dominant phases were observed in the range of the middle frequency (MF from 10 to 1 kHz) for both photocathodes, which gradually decreased and shifted toward high frequencies under negative applied bias. This MF signal could be associated with the charge transport behavior in the photocathode, indicating that the charge transport is promoted as band bending by the applied negative potential due to the inverse relationship between the charge transport time and frequency. In this frequency region, the larger arcs at all applied voltages for the CBS photocathode are determined compared to the BS‐CBS photocathode (Figure S21, Supporting Information). For example, a larger resistance (155 Ω cm^2^) is observed for the CBS photocathode, while the BS‐CBS photocathode shows a relatively smaller resistance (26.2 Ω cm^2^) at 0.6 V_RHE_ (Table S3, Supporting Information). Moreover, the additional peak was only observed in the CBS photocathode at the MHF region, which could be an indicator of the sluggish charge transport in the CBS photocathode. These results support our hypothesis that the large CBM mismatch in the CBS photocathode serves as the recombination center, as manifested by the additional creation of charge trapping capacitance (CPE_MHF_) and resistance (*R*_MHF_). Furthermore, the *J*–*V* curves were also measured under the same conditions for the Bi_2_S_3_ only photoelectrodes in a configuration of Bi_2_S_3_/Pt and Bi_2_S_3_/CdS/TiO_2_/Pt. It was confirmed that only a low anodic current (<0.005 mA cm^−2^) was observed (Figure S22, Supporting Information). This result strongly indicates that the PEC properties of Bi_2_S_3_ have little effect on the PEC performance of the BS‐CBS photocathode.

**Figure 7:**
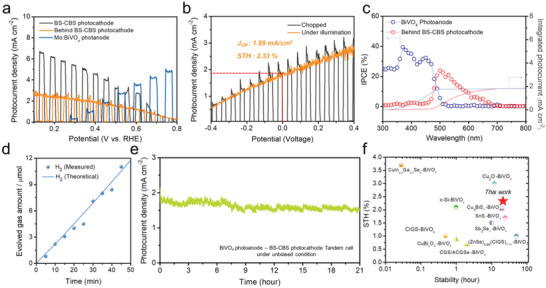
*J–V* curves for a) BS‐CBS photocathode, BiVO_4_ photoanode, and BS‐CBS photocathode behind the BiVO_4_ photoanode and b) BiVO_4_–BS‐CBS tandem cell; *J*_op_ is represented at 0 V. c) IPCE and integrated photocurrent density at 0.5 V_RHE_ (potential for tandem operation) for the photoanode (red) and photocathode behind the photoanode (blue). d) Hydrogen (H_2_) evolution amount–time curves of the unbiased tandem cell. e) Photocurrent density versus time curve for the unbiased tandem cell. The active area of photocathode and photoanode are the 0.15 and 0.1 cm^2^, respectively. f) STH efficiency and stability comparison graphs for BiVO_4_ photoanode–photocathode devices in recent years.

A standalone PEC–PEC tandem device was fabricated by connecting the BS‐CBS photocathode with high onset potential, which is appropriate for D4 tandem, and BiVO_4_‐based photoanode in series, where the electrons (or holes) were transferred from the photoanode to the photocathode (or vice versa). The Mo:BiVO_4_ (*E*_g_ of 2.4 eV) photoanode used here revealed excellent performance (≈5 mA cm^−2^ at 0.8 V_RHE_ and an onset potential of 0.2 V_RHE_ in a K–Pi buffer solution with a pH of 7) as a top light absorber (Figure S23, Supporting Information). Both electrodes were immersed in a K–Pi buffer electrolyte and irradiated with simulated solar light (AM 1.5G) to provide the energy to split water. The operating current density of the overall device was estimated at the intersection of the separately measured *J*–*V* curves of both photoelectrodes in a three‐electrode configuration (**Figure** **7**a). Nearly 2 mA cm^−2^ at 0.5 V_RHE_ was predicted as the operating point, suggesting that sufficient photovoltages (0.9 and 0.98 V from the BS‐CBS photocathode and BiVO_4_ photoanode, respectively) were applied to generate H_2_. The *J–V* curve measurements for the PEC tandem assembled in a two‐electrode configuration were performed to confirm device performance. Figure 7b reveals an operating photocurrent density of 1.89 mA cm^−2^ at 0 V, and the photocurrent value at this voltage represents the unbiased reaction in the two‐electrode mode. The measured unbiased solar H_2_ production current density for the device agrees with the estimated value in Figure 7a. Moreover, the Faradaic efficiency of the tandem cell was close to 100% at a current similar to that of the tandem device (Figure 7d), corresponding to a high *η*_STH_ of 2.33% (Equation (5)).

(5) $$H_{\mathrm{STH}}=\left(J_{\mathrm{op}}\times 1.23\times \eta_{F}\right)/P$$

where *P* is the power of the illuminated light, *J*_op_ represents the photocurrent density operation of a tandem cell, and *η*_F_ represents the Faradic efficiency. The achieved *η*_STH_ efficiency is remarkably high for unbiased tandem cells.

The photon absorption and conversion capability of the Mo:BiVO_4_–BS‐CBS tandem cell was verified through IPCE measurements. The IPCE spectra (Figure 7c) were measured under the operating bias (0.5 V_RHE_) for both electrodes. From the analogy of the absorption wavelength range from the photoanode and photocathode, the integrated IPCE photocurrent density for a rear photocathode was reduced by 52% compared to that of the single photocathode (Figure S24, Supporting Information). Nevertheless, the integrated photocurrent density of the rear photocathode was observed to be as high as 1.8 mA cm^−2^, which coincides with the operating photocurrent density of the tandem cell. The photocurrent of the overall device under unbiased conditions remained constant for over 21 h (Figure 7e). In comparison with previously reported results for photocathode–photoanode tandem cells, it was found that the efficiency of the BiVO_4_–BS‐CBS tandem cell (2.33% STH) was not the highest when compared to those of BiVO_4_–CIGS (3.7%) and BiVO_4_–Cu_2_O (3.0%). However, bearing in mind the low cost of the materials, facile fabrication, and short history, the performance of the BS‐CBS‐based tandem cell (STH efficiency of 2.33% and stability of over 21 h) can be considered as a significant benchmark for PEC tandem cells (Figure 7f).

## 3 Conclusions

In conclusion, an unbiased tandem cell combined with a BS‐CBS photocathode and Mo:BiVO_4_ photoanode produced a high STH efficiency of 2.33%. This benchmark value was achievable owing to the BS‐CBS photocathode having a high photocurrent as well as inherent high onset potential of 0.9 V_RHE_ from its wide *E*_g_. In situ growth of the Bi_2_S_3_–CBS mixed‐phase nanorods was successfully achieved via an innovative solution process by simply modulating the initial Bi/Cu ratio in the precursor ink. The unique film formation mechanism showed that a precursor ink containing excess dissolved Bi beyond the stoichiometric amount led to simultaneous production of elongated 1D nanorods atop the Cu_3_BiS_3_ planar film. The synergetic effects of enhanced light absorption and excellent charge transport of the BS‐CBS photocathode allowed three‐fold ABPE enhancement compared to that of the CBS photocathode, enabling our BS‐CBS photocathode to provide the highest PEC performance among reported Cu_3_BiS_3_‐based photocathodes. Furthermore, by combining the material with a Mo:BiVO_4_ photoanode, unbiased overall water splitting was achieved with an impressive efficiency and a high stability of over 21 h. Given the relatively short history of these photoelectric materials, the performance indicators including stability, onset potential, and photocurrent density obtained herein are comparable with those of the highly investigated photocathodes. Clearly, it is indicated that our findings represent an important demonstration of a PEC tandem and will lead to better efficiency of Cu_3_BiS_3_‐based devices in the near future.

## 4 Experimental Section

### Preparation of Cu_3_BiS_3_ Films

To prepare the precursor solutions, thiourea (Sigma Aldrich, 99%), copper nitrate trihydrate (1.95, 2.6, or 2.9 mmol, Sigma Aldrich, 99%), and bismuth nitrate pentahydrate (1.59, 1.06, or 0.79 mmol, Sigma Aldrich, 98%) were weighed and sequentially dissolved in 2‐mercaptoethanol (7.5 mL, Sigma Aldrich, 99%). Cleansing of the substrate was conducted by ultrasonication in acetone and ethanol for 10 min, followed by UVO treatment for 15 min. The prepared inks were spin coated onto evaporated Au(70 nm)/FTO glass substrates at 2500 rpm for 30 s. Once the films were deposited, they were sequentially annealed at 180 and 300 °C in a N_2_‐filled glovebox for 1 min. The spin‐coating process was repeated 6 times for each ink.

### Preparation of Cu_3_BiS_3_ Photocathode

CdS layers were deposited by chemical bath deposition (CBD). The CBD process was performed by immersing the samples in a bath solution. The solution was prepared using CdSO_4_, thiourea (Sigma Aldrich, 99%), deionized water, and NH_4_OH (Duksan, 28 wt%, Korea). The process was performed for 11 min and 30 s at 60 °C, and the sample was then rinsed using deionized water. TiO_2_ layers were deposited via atomic layer deposition (Lucida D 100, NCD Inc.) using tetrakis(dimethylamido) titanium (TDMAT, Easychem, Korea) and H_2_O as the Ti and O sources, respectively. The TiO_2_ layer was deposited for 600 cycles at 120 °C. A 108 Auto Sputter Coater (Ted Pella, Reding, CA, USA) was used to deposit the Pt cocatalyst for 120 s under 10 mA current and 0.1 mbar pressure of Ar.

### Fabrication of Mo:BiVO_4_ Photoanodes

The FTO glass substrates were cleaned and placed in an e‐beam evaporator with carbon crucibles filled with SnO_2_ sources (Taewon Scientific Co., 99.99%). After evacuating the chamber to a pressure of 2 × 10^−6^ torr, the SnO_2_ adhesion layers were deposited on the FTO glass substrates. By glancing angle deposition (glancing angle of 85°, rotation speed of 80 rpm), the SnO_2_ nanorods were formed, followed by annealing for 2 h at 550 °C. With an Ag/AgCl (3 m NaCl) reference electrode and a Pt counter electrode, BiOI was electrodeposited on the SnO_2_ nanorods in a three‐electrode cell. To prepare the plating solution, 20 mL of ethanol (Daejung, 99.9%) with 46 mm p‐benzoquinone (JUNSEI, 98%) and 50 mL of an aqueous solution containing 400 mm KI (Daejung, 99.5%), 15 mm Bi(NO_3_)_3_∙5H_2_O (Junsei, 98%), and 30 mm lactic acid (Aldrich, 85%) was prepared. Following mixing of the plating solution for 30 min, nucleation and growth of the BiOI were carried out at −0.35 V versus Ag/AgCl for 20 s, followed by growth of BiOI at −0.10 V versus Ag/AgCl for 360 s.

A mixture of 10 µL of an aqueous solution containing 100 mm Na_2_MoO_4_ (Daejung, 98.5%) and 5 mL of DMSO (Kanto, 98%) containing 200 mm VO(acac)_2_ (Aldrich, 98%) was prepared for conversion into Mo‐doped BiVO_4_ (Mo: BiVO_4_). After annealing (in air, 450 °C), the BiOI/SnO_2_ nanorods were soaked in 50 µL of the mixing solution for 2 h, followed by removal of the residual V_2_O_5_ in a 1 m NaOH solution (Daejung, 99%) for 10 min. Carbon crucibles filled with NiFe alloy pellets (50 wt%:50 wt%) were applied for e‐beam evaporation. Deposition of the NiFe oxygen evolution catalyst on Mo:BiVO_4_/SnO_2_ nanorods occurred at a rate of 0.2 Å s^−1^.

### Characterizations

The surface morphologies of the films were examined using a field‐emission SEM (JSM‐7001F, JEOL, Japan) with a 15 kV accelerating voltage. The phases of the samples were determined by XRD (MiniFlex 600, Rigaku, Japan) using Cu K*α* radiation (*λ* = 0.15406 nm). Raman mapping images and spectra were collected with a confocal Raman microscope (Alpha 300 Apyron, WITec, Germany). TEM (Jeol, JEM‐F200, JED‐2200 detector, Japan) was carried out to analyze the phases of the films. Under He I radiation (21.2 eV), UPS (ThermoFisher Scientific, UK) was used to determine the energy levels of the semiconductors. The secondary‐electron cutoff (*E*_cutoff_) is derived by extrapolating the linear part of the binding energy edge to the secondary electron. The Fermi level (*E*_f_) of each material was calculated as follows: *E*_f_ = *E*_cutoff_ − 21.2 eV (under He I radiation). The valence band edge (*E*_edge_) represents the difference between *E*_VBM_ and *E*_f_ for each material. Using the *E*_edge_ values, the definite VBM levels of the samples were calculated as follows: *E*_VBM_ = *E*_edge_ + *E*_f_. To compare the electrical conductivities of the samples, c‐AFM measurements (SPA 400, Seiko Instruments, Inc., Japan) were obtained using a Rh‐coated cantilever with an area of 2 µm × 2 µm. TRPL study was carried out using a confocal microscope (MicroTime‐200, Picoquant, Germany) with a 40× (air) objective. The lifetime measurements were performed at the Korea Basic Science Institute (KBSI), Daegu Center, Korea. A single‐mode pulsed diode laser (470 nm with a pulse width of ≈30 ps and an average power of ≈250 µW operating with 40 MHz repetition rate) was used as the excitation source. A dichroic mirror (490 DCXR, AHF), a longpass filter (HQ500lp, AHF), a 150 µm pinhole, a longpass filter (FEL0550, Thorlabs), and a single‐photon avalanche diode (PDM series, MPD) were used to collect emissions from the samples. A time‐correlated single‐photon counting system (PicoHarp‐300, PicoQuant GmbH, Germany) was used to count the emitted photons. The optical properties were determined using a UV–vis spectrophotometer coupled to an integrating sphere (V‐670, JASCO, Easton, MD, USA).

### PEC Measurements

Using a potentiostat (SI 1287, Solartron, Leicester, UK), PEC measurements of the Cu_3_BiS_3_ photocathode were performed with a conventional three‐electrode configuration in a 0.5 m K‐Pi solution (pH 7). An Ag/AgCl/KCl (saturated) electrode and a coiled Pt wire were used as the reference and counter electrodes, respectively. Simulation of sunlight and 1‐sun calibrations were conducted using a commercial AM 1.5G solar simulator and a Si reference cell (Newport Corporation, USA). The PEC measurements for the tandem devices were performed in a 0.5 m K‐Pi solution (pH 7). For all PEC measurements, the applied potentials were converted to RHE values using the following equation: *E*_RHE_ = *E*_Ag∕AgCl_ + 0.059 pH + 0.197. EIS measurements were obtained using the potentiostat along with a frequency analyzer (1260, Solartron, Leicester, UK). The series and polarization resistances of the photocathodes were measured in the frequency range of 50 kHz to 0.5 Hz with an AC amplitude of 10 mV under solar irradiation (AM 1.5G). The IPCE and IMVS measurements were performed using an electrochemical workstation (Zennium, Zahner, Germany) and a potentiostat (PP211, Zahner, Germany) with a monochromatic light source (TLS03, Zahner, Germany). IMVS was conducted in the open‐circuit condition with 10% perturbation of the light intensity. The frequency of light modulation was swept from 100 kHz to 10 Hz. To analyze the evolution of H_2_, a pulsed discharge detector and a molecular sieve column were used in the chromatography (6500GC system, YL Instrument, Anyang, Korea). Rubber bulkheads were used to seal all the connections of the quartz reactor to prevent gas leakage.

### Statistical Analysis

Data were presented as means ± standard deviation regarded as statistically significant. The statistical results of PEC performance for the BS‐CBS photocathode in Figure S25, Supporting Information were applied on the basis of the performance for 7 different BS‐CBS photocathodes.

## Conflict of Interest

The authors declare no conflict of interest.

## Supporting information

Supporting Information

## Data Availability

The data that support the findings of this study are available from the corresponding author upon reasonable request.;
