# Supplementary material for: Bi2S3‐Cu3BiS3 Mixed Phase Interlayer for High‐Performance Cu3BiS3‐Photocathode for 2.33% Unassisted Solar Water Splitting Efficiency
Source: Adv Sci (Weinh). 2023 Jan 16;10(6):2206286. doi: 10.1002/advs.202206286 (PMC9951361; doi:10.1002/advs.202206286)
Supplement: Supplementary file 1 — Supporting Information [file ADVS-10-2206286-s001.pdf]

Supporting Information

Bi_2S_3-Cu_3BiS_3 Mixed Phase Interlayer for High-Performance Cu_3BiS_3-Photocathode for 2.33% Unassisted Solar Water Splitting Efficiency

Subin Moon[‡1], *Jaemin Park*[‡1], *Hyungsoo Lee*[1], *Jin Wook Yang*[2], *Juwon Yun*[1], *Young Sun Park*[1], *Jeongyoub Lee*[1], *Hayoung Im*[1], *Ho Won Jang*[2]*, *Wooseok Yang*[3,4]*, *and Jooho Moon*[1]*

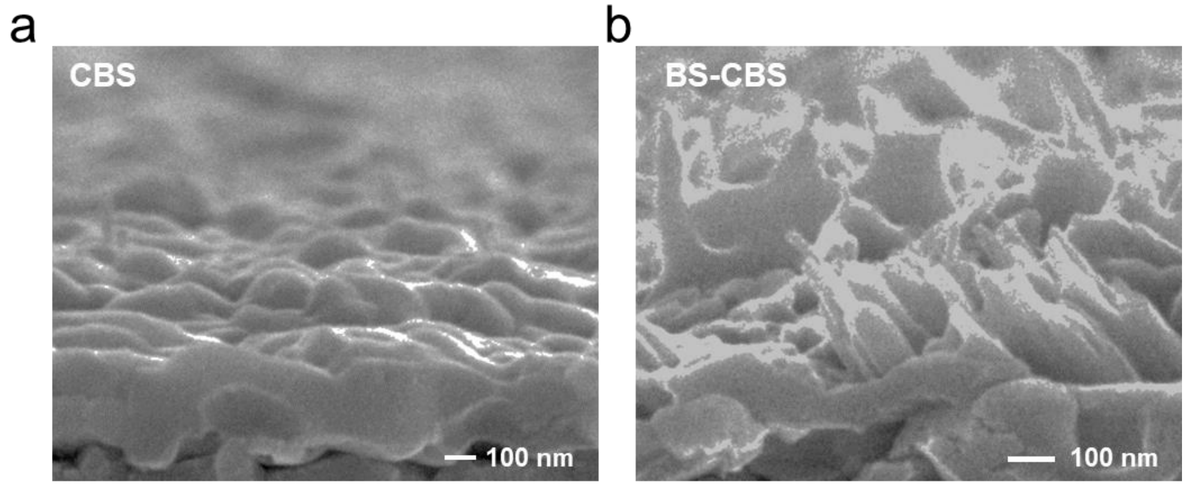

Figure S1. Cross-sectional scanning electron microscopy (SEM) images of a) CBS and b) BS-CBS films.

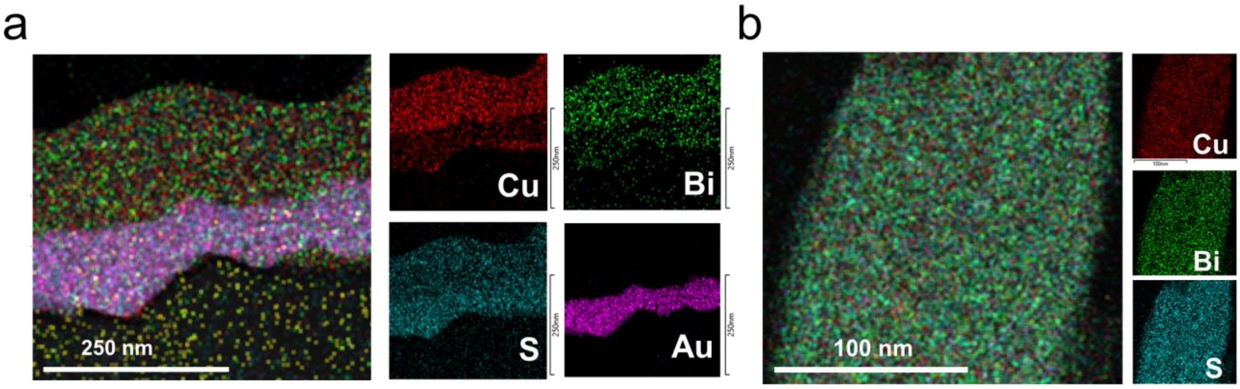

Figure S2. TEM-EDS elemental mappings of a) CBS and b) BS-CBS films.

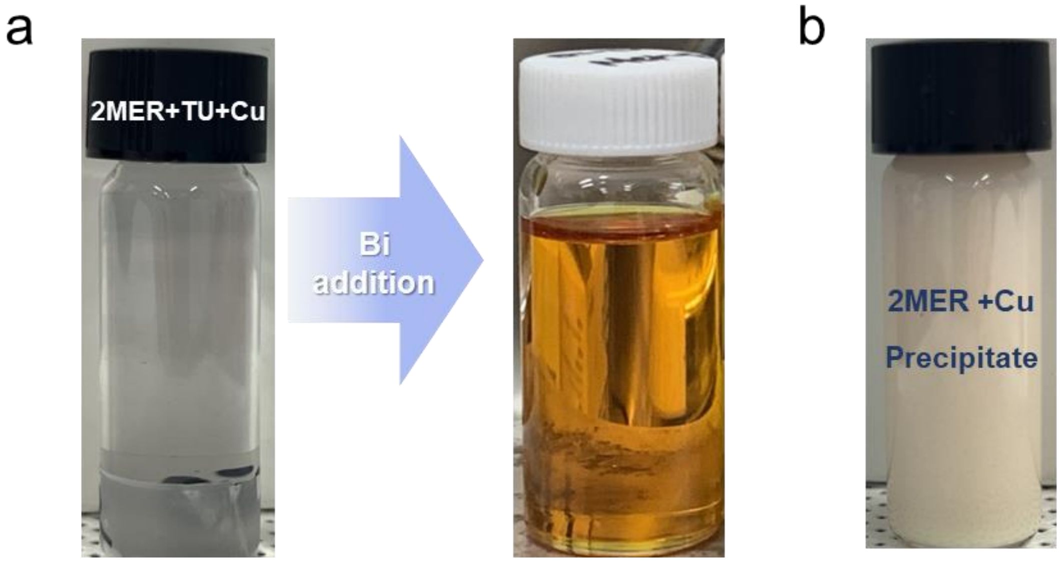

Figure S3. Photographs of precursor inks containing a) 2MER+TU+Cu and 2MER+TU+Cu+Bi and b) 2MER+Cu.

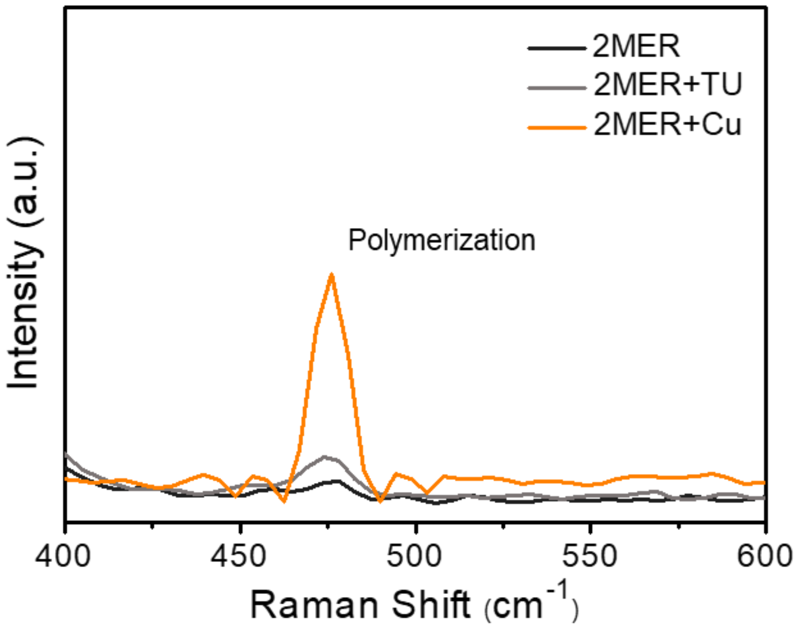

Figure S4. Raman spectra corresponding to precipitation by adding Cu precursor to 2MER.

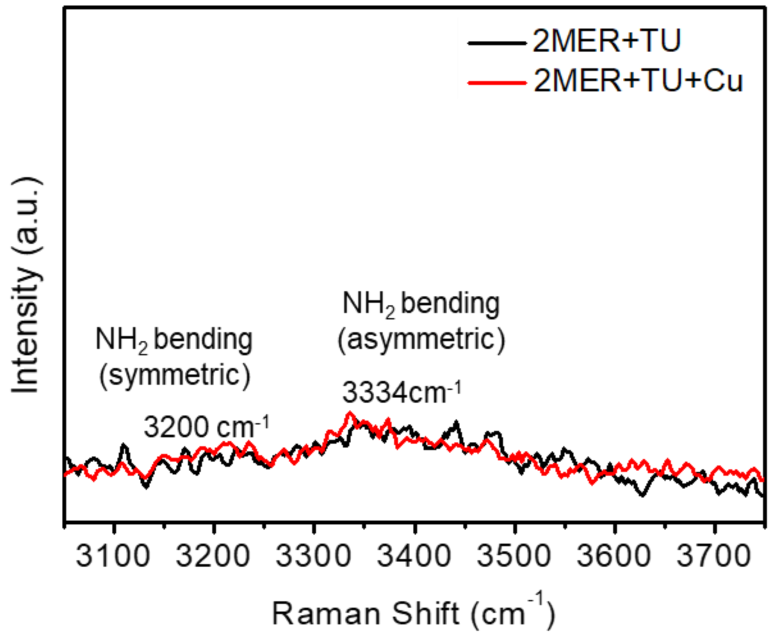

Figure S5. Raman spectra corresponding NH_2 bending for inks containing 2MER+TU and 2MER+TU +Cu.

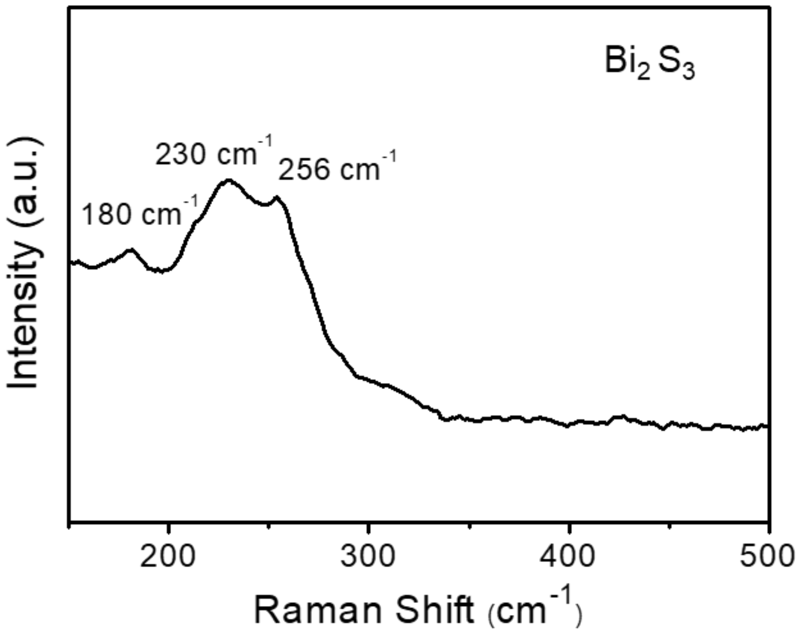

Figure S6. Raman spectrum for the Bi_2S_3 film.

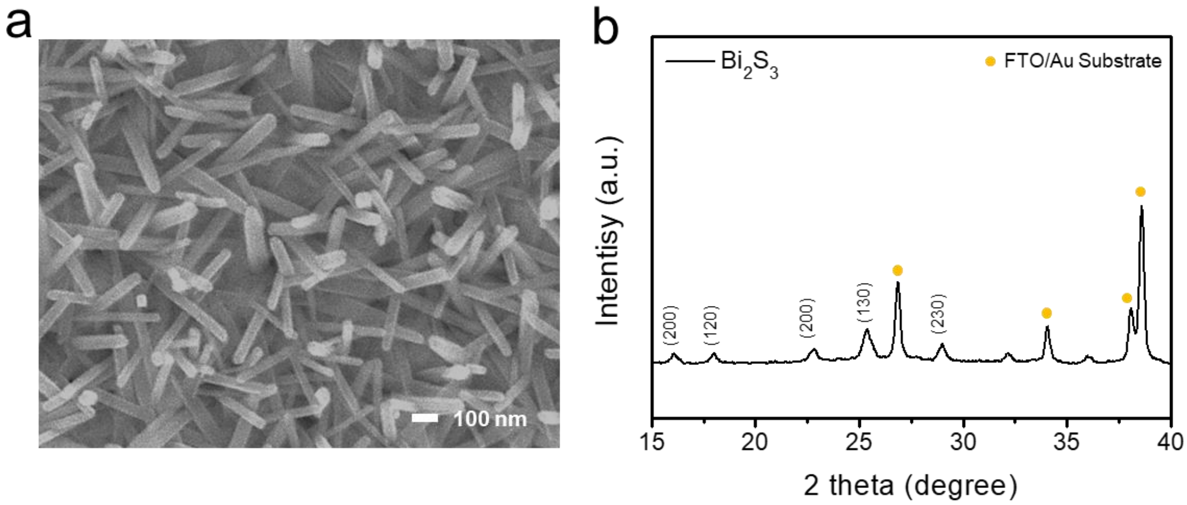

Figure S7 a) Top-view SEM image and b) XRD pattern for Bi_2S_3 film.

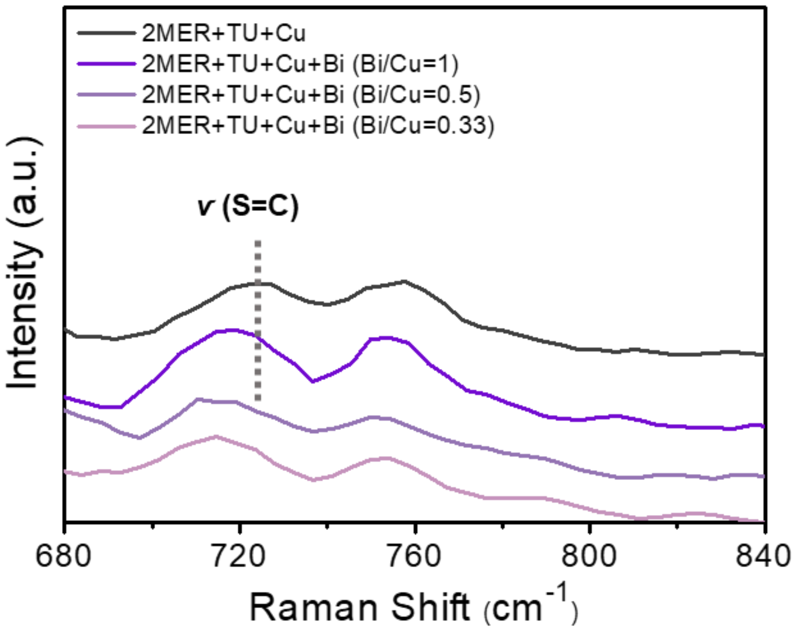

Figure S8. Raman spectra for the C=S bonding of inks with different Bi/Cu ratios.

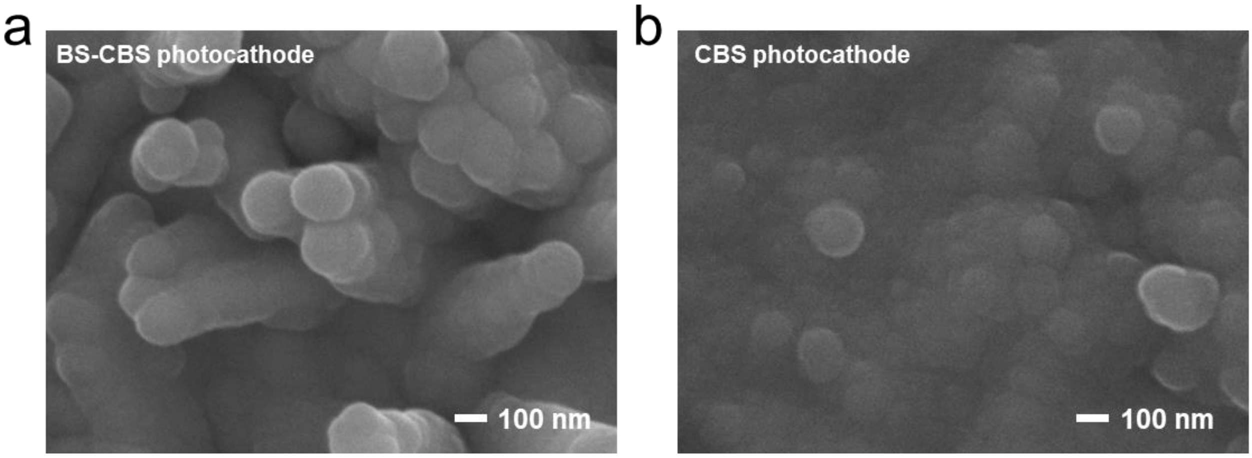

Figure S9. Top-view SEM images of a) BS-CBS/CdS/TiO_2/Pt and b) CBS/CdS/TiO_2/Pt photocathodes.

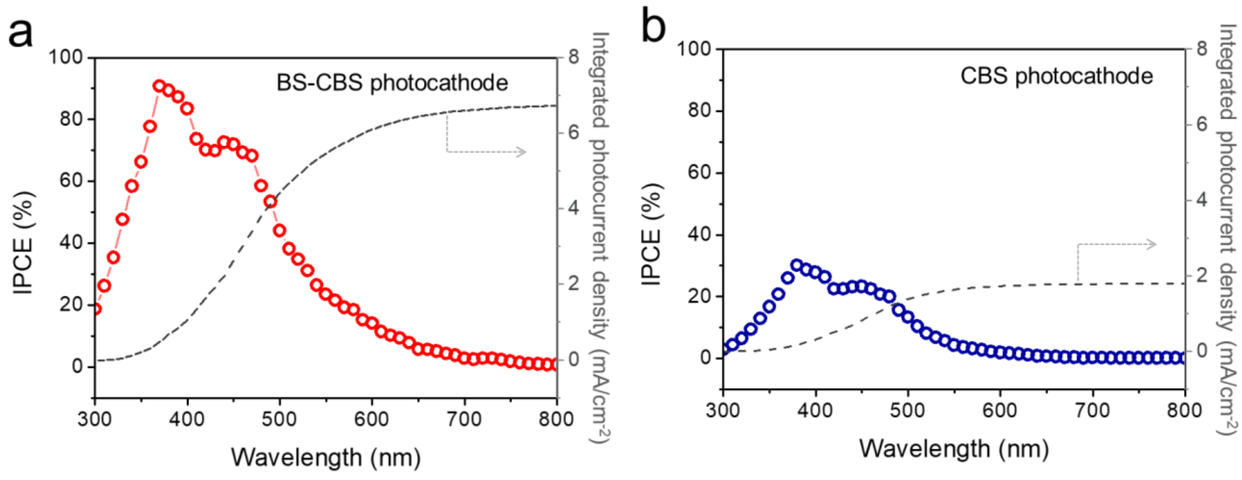

Figure S10. IPCE spectra of a) BS-CBS/CdS/TiO_2/Pt and b) CBS/CdS/TiO_2/Pt photocathodes under solar simulated AM 1.5G irradiation in 0.5 M K-Pi electrolyte (pH 7).

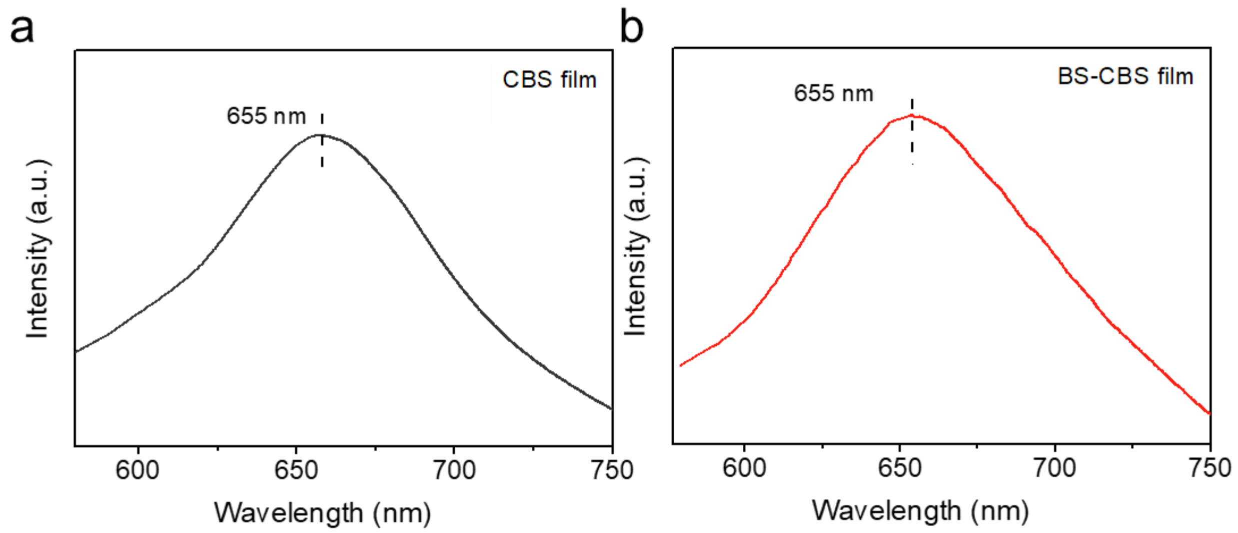

Figure S11. PL spectra of a) CBS and b) BS-CBS films.

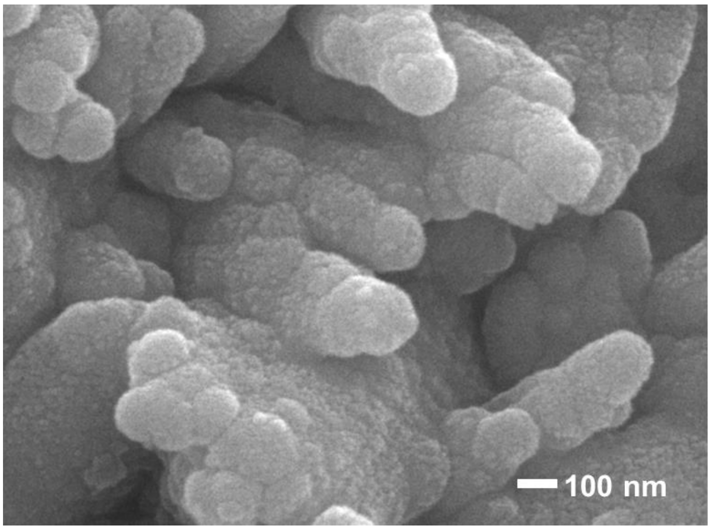

Figure S12. A top-veiw SEM image BS-CBS photocathode after PEC operation.

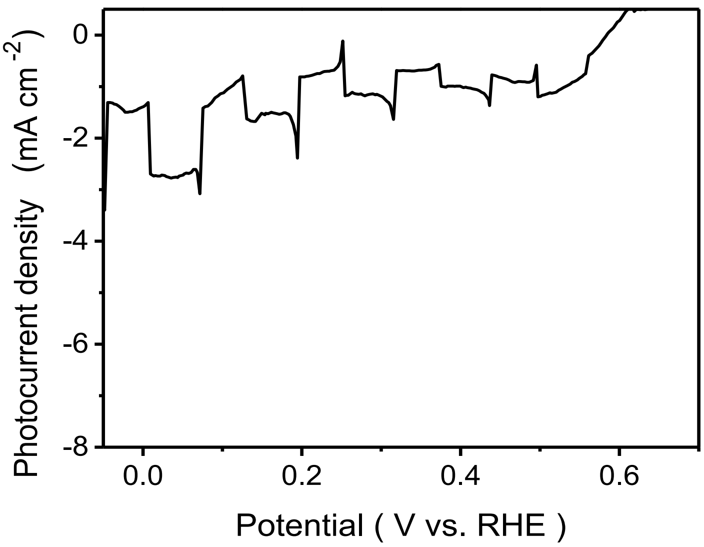

Figure S13. J-V curve for the BS-CBS photocathode after stability test for 20 h under 0 V_{RHE} in 0.5 M K-Pi electrolyte (pH 7).

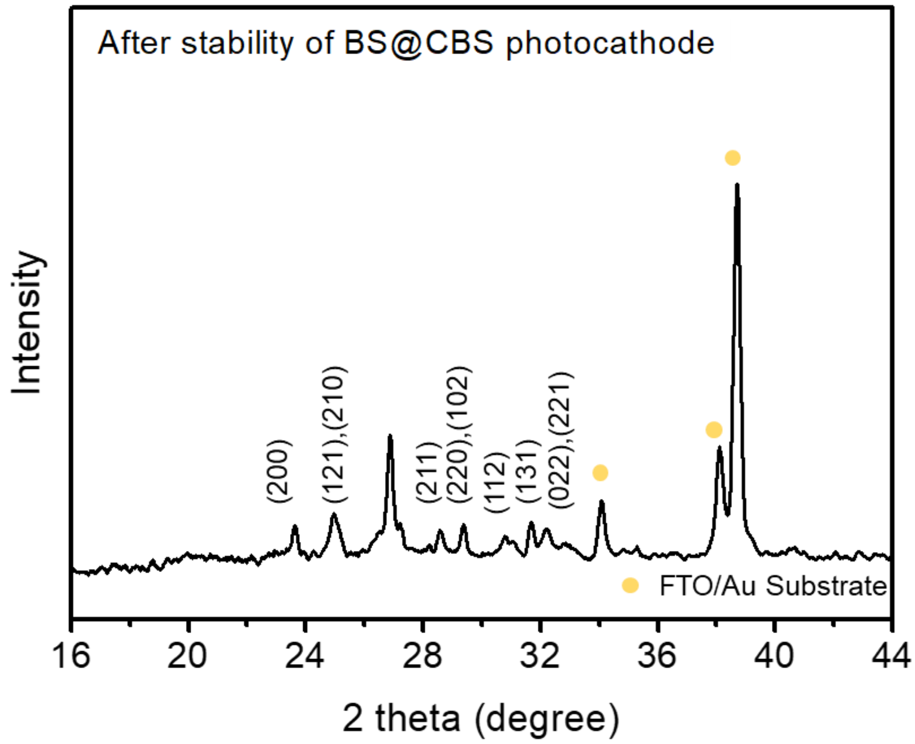

Figure S14. The XRD pattern of BS-CBS photocathode after PEC operation for 20h.

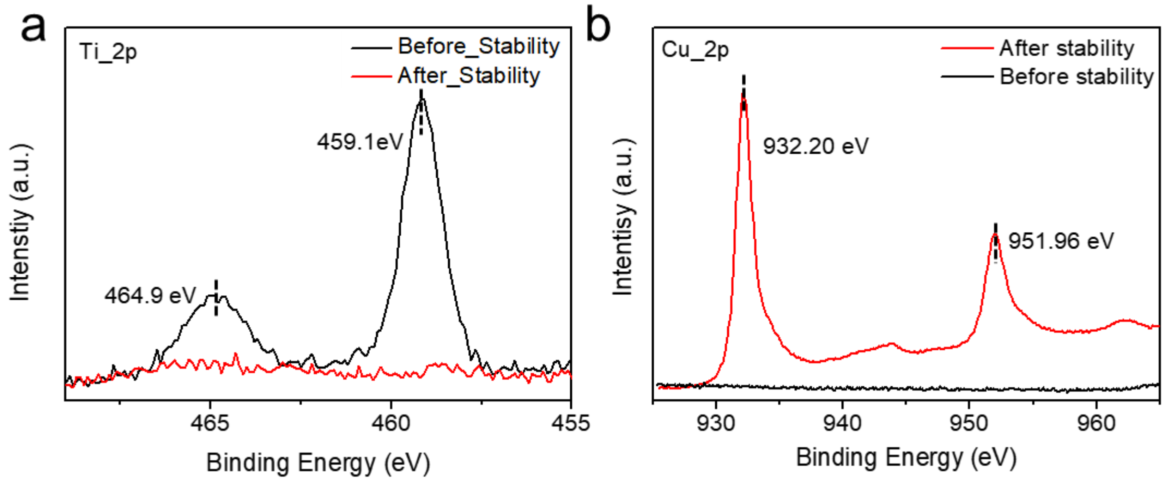

Figure S15. XPS spectra for a) Ti and b) Cu chemical states of before-stability test and after-stability test.

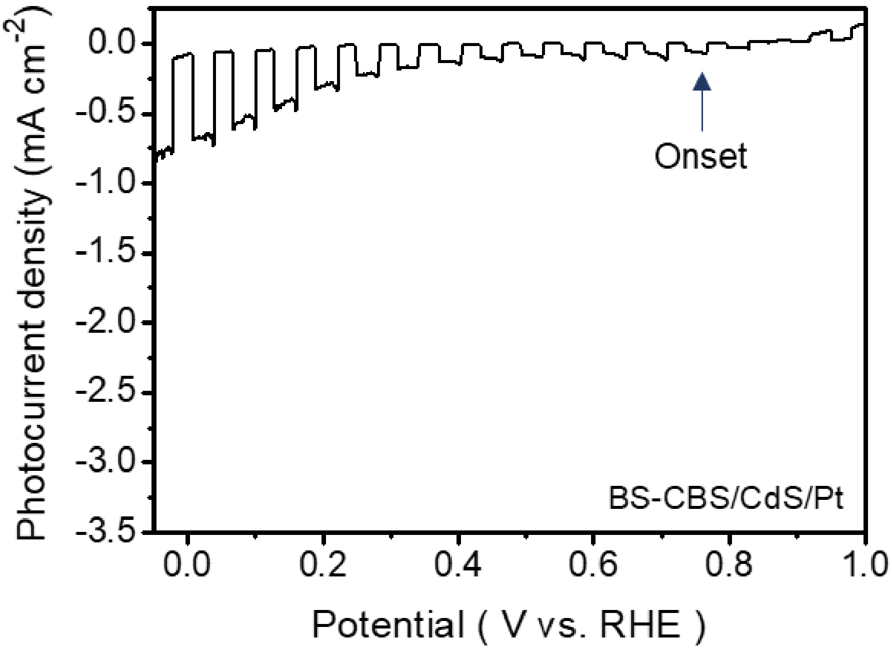

Figure S16. J-V curve for a) BS-CBS/CdS/Pt photocathode.

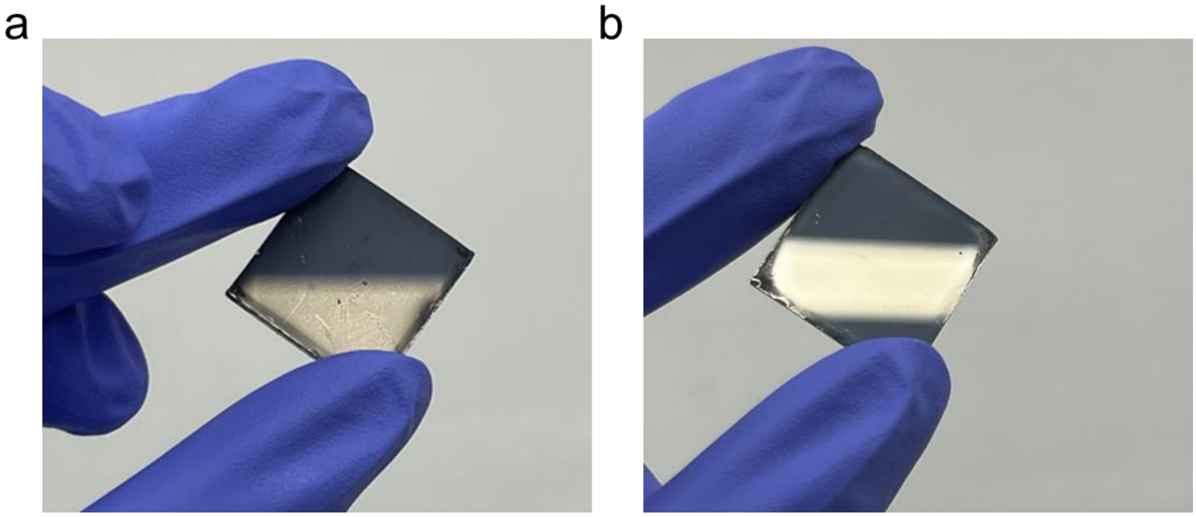

Figure S17. Photographs of a) BS-CBS and b) CBS films.

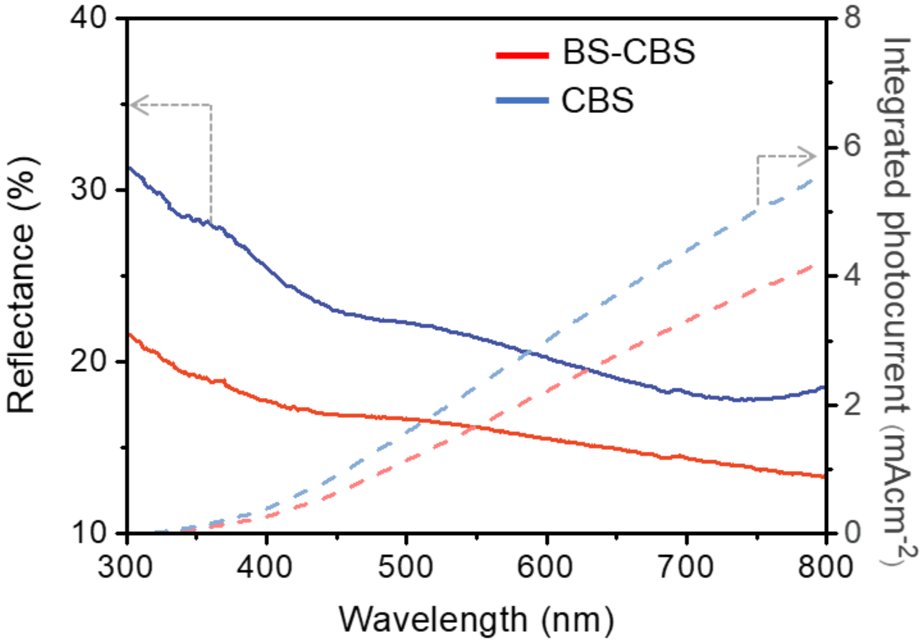

Figure S18. Total reflectances of the CBS and BS-CBS films as well as their integrated photocurrent densities, assuming that the reflected light is absorbed with 100% photon-to-current conversion efficiency. The total reflectance was obtained as the sum of the specular and diffuse reflectances.

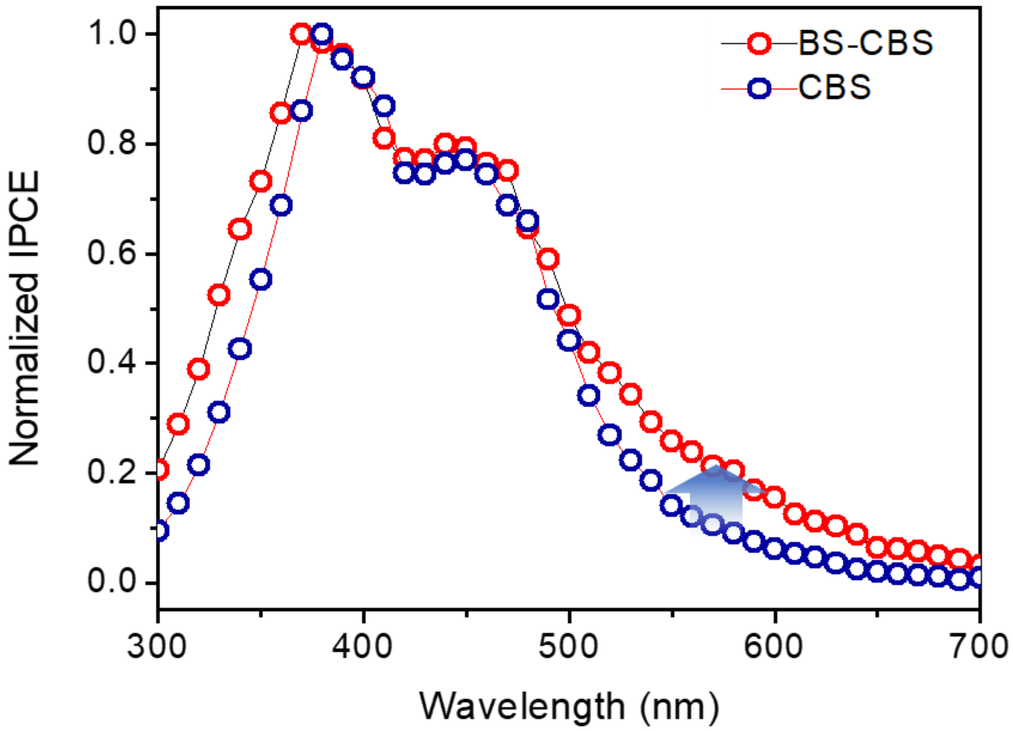

Figure S19. Normalized IPCE spectra of the BS-CBS/CdS/TiO_2/Pt and CBS/CdS/TiO_2/Pt photocathodes under solar simulated AM 1.5G irradiation in 0.5 M K-Pi electrolyte (pH 7).

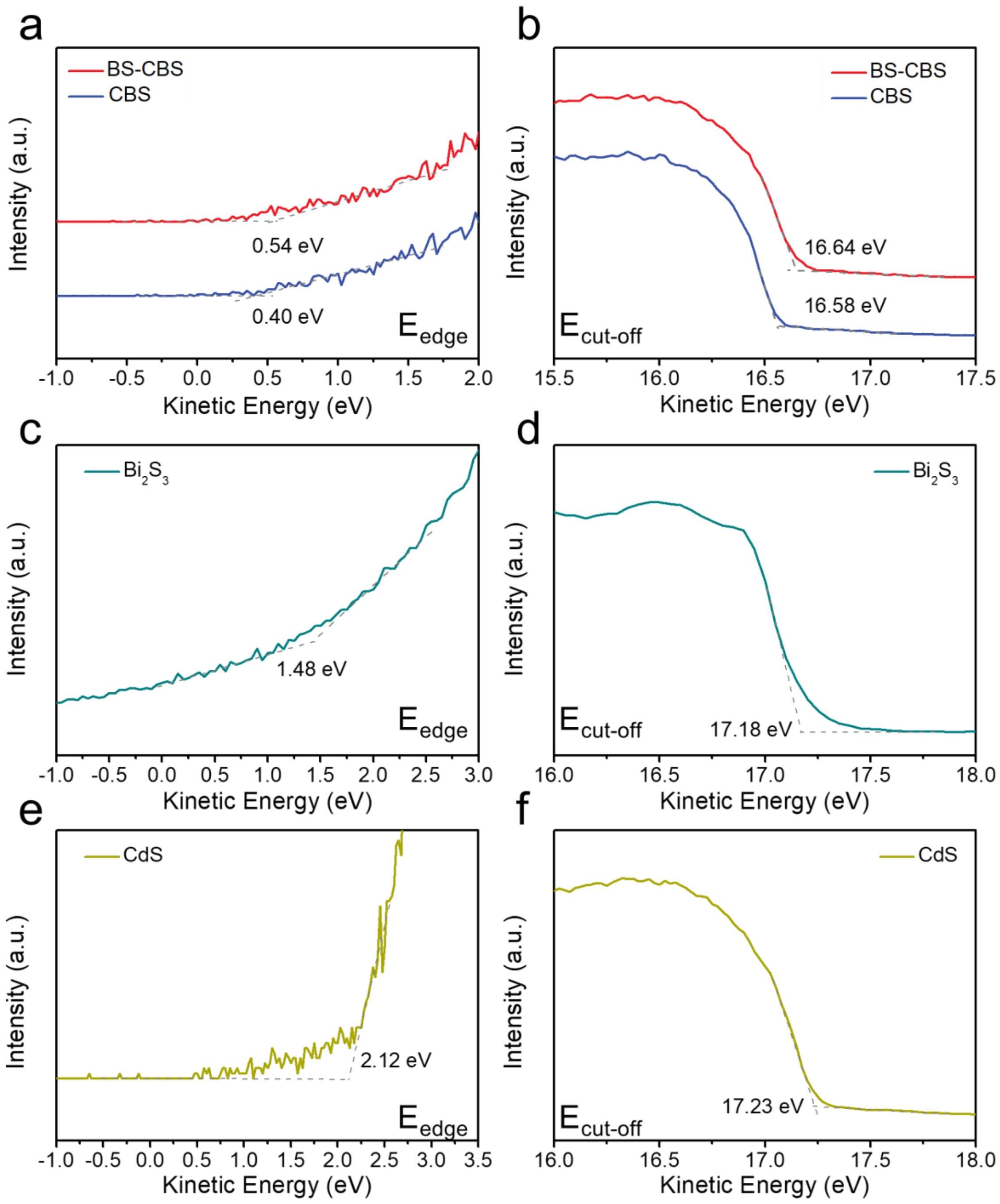

Figure S20. Normalized UPS data of the valence band edges for a) CBS, BS-CBS, c) Bi_2S_3, and e) CdS. Secondary electron cut-off regions for the b) CBS, BS-CBS, d) Bi_2S_3, and f) CdS.

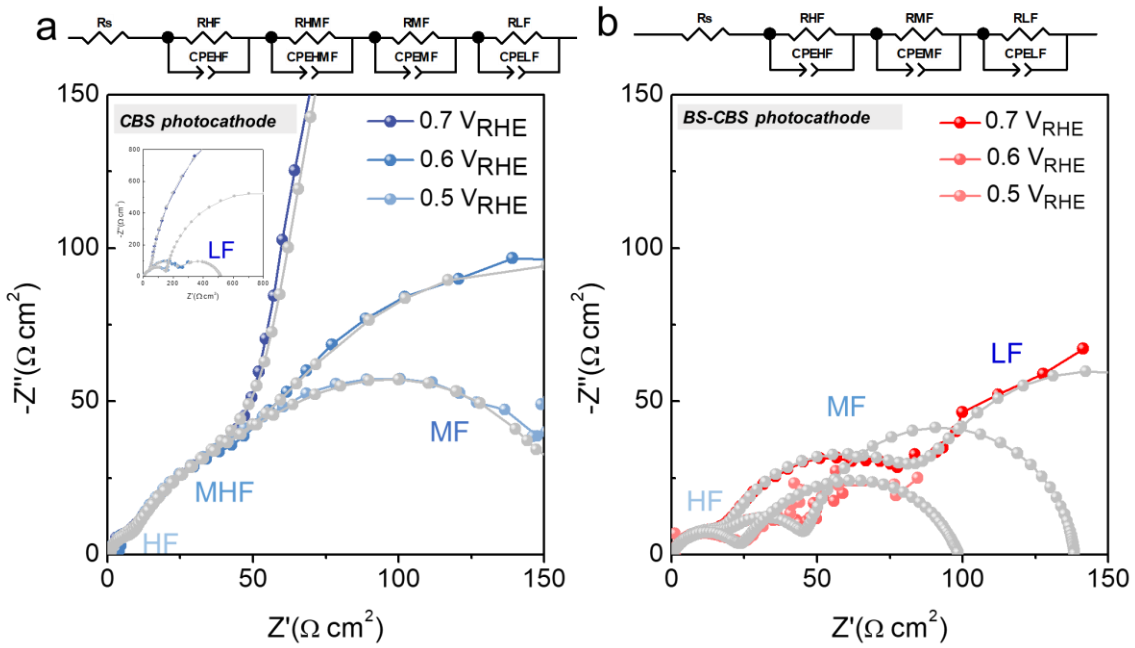

Figure S21. Nyquist plots obtained from the EIS spectra for the a) CBS and b) BS-CBS photocathodes in 0.5 M K-Pi electrolyte (pH 7).

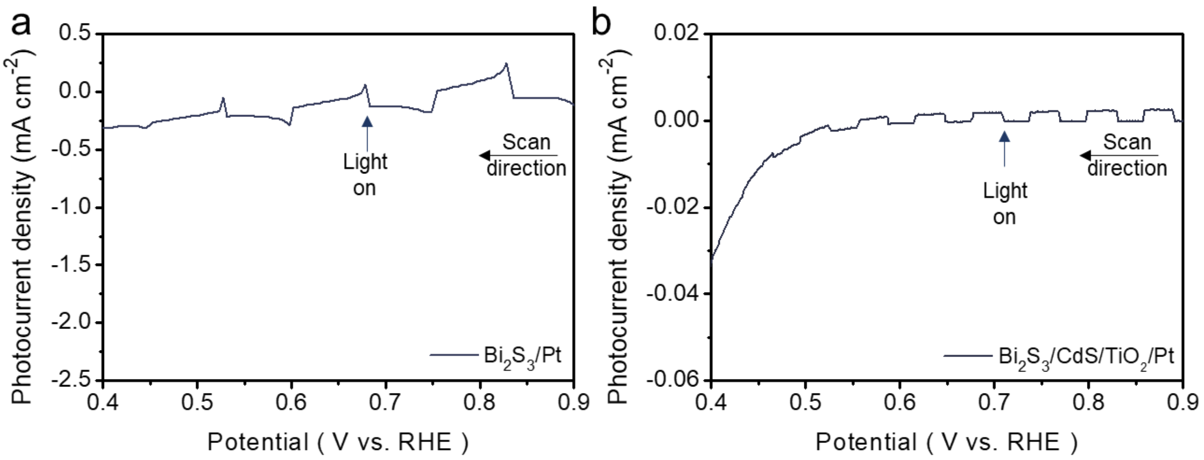

Figure S22. a) J-V curve for Bi_2S_3/Pt and b) for Bi_2S_3/CdS/TiO_2/Pt.

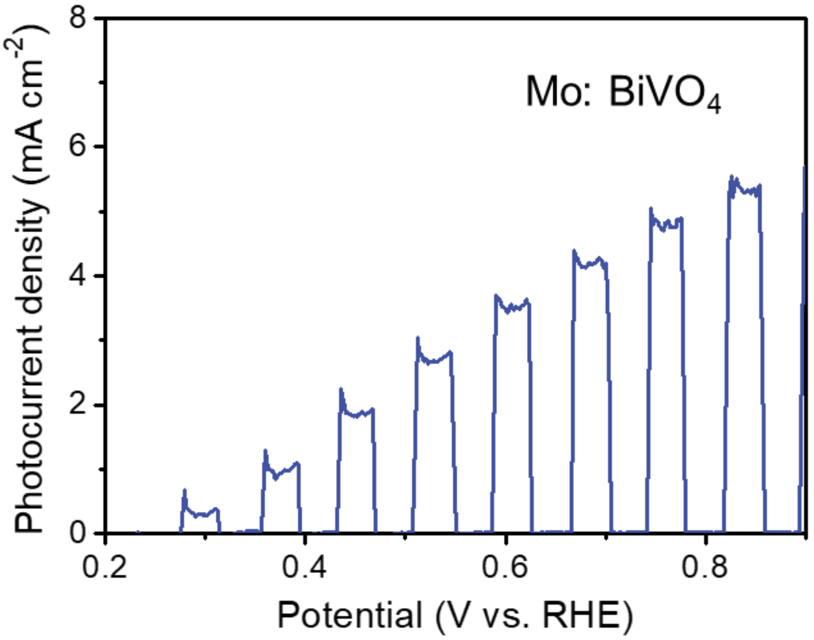

Figure S23. J-V curves for the Mo:$BiVO_4$ photoanode under solar simulated AM 1.5G irradiation in 0.5 M K-Pi electrolyte (pH 7).

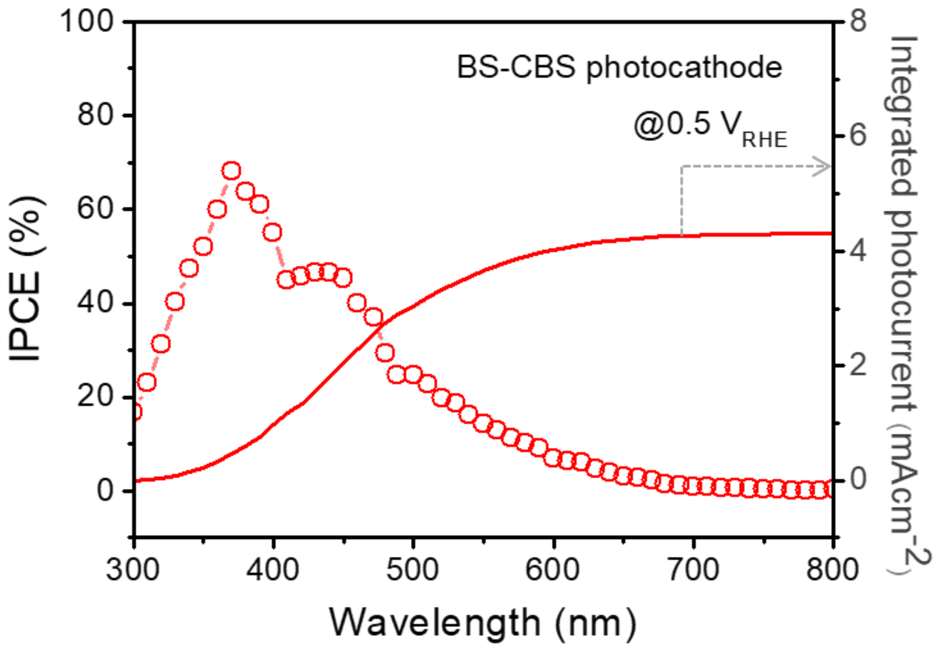

Figure S24. IPCE and integrated photocurrent density of BS-CBS photocathode at 0.5 V_{RHE}.

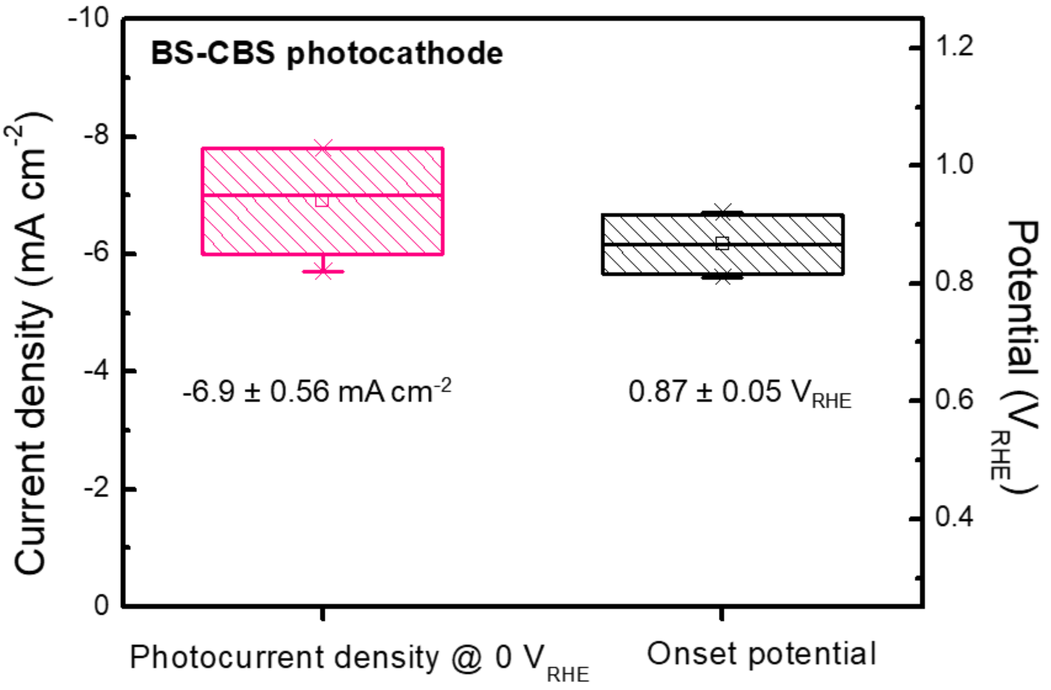

Figure S25. The PEC distribution for the photocurrent density and onset voltage of the 7 different BS-CBS photocathodes with sample size of 0.15 cm^2.

Table S1. The atomic ratio determined by ICP-MS results for the films with Bi/Cu=1, Bi/Cu=0.5, and Bi/Cu=0.33 inks.

Element	Cu (at%) (atomic ratio)	Bi (at%) (atomic ratio)	S (at%) (atomic ratio)
Bi/Cu = 1 film	35 ± 4.1 (1)	26 ± 0.89 (1)	43 ± 0.39 (1)
Bi/Cu = 0.5 film	39 ± 0.84 (0.77)	20 ± 0.78 (0.62)	41 ± 0.18 (0.29)
Bi/Cu = 0.33 film	45 ± 0.56 (1.9)	15 ± 0.26 (1.35)	40 ± 0.57 (0.82)

Table S2. TRPL fitting data of the lifetime components (τ) and weight fraction (A) obtained from the TRPL decay curves of the Au/CBS and Au/BS-CBS samples.

	BS-CBS	CBS
A_1 (%)	53.5	54.3
A_2 (%)	45.97	45.06
A_3 (%)	0.45	0.57
τ_1 (ns)	0.0241	0.0216
τ_2 (ns)	0.2287	0.2357
τ_3 (ns)	2.8	3.5
$\tau_{average}$ (ns)	**0.451**	**0.7**

Table S3. Area-specific resistance values and CPEs obtained by deconvolving the EIS spectra at 0.6 V_{RHE}.

Photocathode	Rs (Ω cm^2)	R_{HF} (Ω cm^2)	CPE_{HF} (FS^{n-1} cm^2)	R_{MHF} (Ω cm^2)	CPE_{MHF} (FS^{n-1} cm^2)	R_{MF} (Ω cm^2)	CPE_{MF} (FS^{n-1} cm^2)	R_{LF} (Ω cm^2)	CPE_{LF} (FS^{n-1} cm^2)
BS-CBS	0.25	20	2.8×10^{-5} (n=0.72)	N/A	N/A	26.2	0.00012 (n=0.86)	92	0.0039 (n=0.93)
CBS	1	7	10×10^{-5} (n=0.96)	55	1.4×10^{-5} (n=0.82)	155	1.32×10^{-5} (n=0.982)	300	0.00099 (n=0.7)
